# Sequential embryo transfer efficacy in enhancing pregnancy outcomes: a systematic review and meta-analysis

**DOI:** 10.1007/s10815-025-03487-5

**Published:** 2025-05-21

**Authors:** Noran Magdy Shalma, Ahmed M. Talaia, Mohamed R. Abdelraouf, Mostafa Abdullah Alsharabasy, Ayah Abdulgadir, Nada K. Abdelsattar, Mohamed Abd-ElGawad

**Affiliations:** 1 Faculty of Medicine, Tanta University, Tanta, Egypt; 2 Faculty of Medicine, Alexandria University, Alexandria, Egypt; 3 Faculty of Medicine, Zagazig University, Zagazig, Egypt; 4 Faculty of Medicine, University of Khartoum, Khartoum, Sudan; 5 Faculty of Medicine, Fayoum University, Fayoum, Egypt

**Keywords:** Sequential embryo transfer, Cleavage transfer, IVF

## Abstract

**Background:**

Sequential embryo transfer (SEQET) can increase the availability of embryos at various stages within the implantation window, thus improving the outcomes of assisted conception. This research seeks to analyze and synthesize clinical information on the influence of SEQET on in vitro fertilization (IVF) embryo transfer outcomes.

**Methods:**

The literature search was done through four databases, which are Pubmed, Web of Science, Scopus, and Cochrane. The inclusion criteria are clinical trials or observational studies comparing sequential embryo transfer (cleavage and blastocyst) to single-day embryo transfer (cleavage or blastocyst) in women undergoing IVF. Data was collected from the included studies and analyzed by RevMan software.

**Results:**

Twenty-three studies fulfilled the criteria for inclusion in this study. We found that SEQET showed significant improvement in clinical and chemical pregnancy rates (*P* < 0.00001) in comparison to the cleavage embryo transfer (CET) group. Moreover, implantation rates (*P* = 0.002) and live births (*P* = 0.006) were significantly greater. In comparing SEQET to blastocyst transfer, SEQET was associated with a significant increase in the clinical pregnancy rate (*P* = 0.003).

**Conclusion:**

This research discovered that sequential embryo transfer significantly enhanced live birth, clinical pregnancy, chemical pregnancy, and implantation rates compared to cleavage transfer. SEQET also improved clinical pregnancy rates compared to blastocyst transfer. However, there was no significant difference between the two groups in terms of live birth, implantation rates, or miscarriages.

**Supplementary Information:**

The online version contains supplementary material available at 10.1007/s10815-025-03487-5.

## Introduction

Steptoe and Edwards were the first to report in vitro fertilization (IVF) success in 1978, which represented a turning point and a promising treatment for infertile couples [1]. However, a documented implantation success rate of 25–40% usually requires repeating the procedures, which are not feasible for most couples [2]. Moreover, about 15% of patients suffer from repeated implantation failure (RIF). RIF is defined as the inability of a good-quality embryo to implant in three different cycles [3, 4]. RIF represents a challenge for women undergoing ART. It constitutes a financial and psychological burden [5, 6]. Thus, it is essential to find an effective strategy to enhance pregnancy outcomes in these patients. Theoretically, the success of implantation depends on the quality of the transferred embryo and the receptivity of the uterine lining. Failure of implantation is either due to a defect in one of these two factors or due to immunological or multiple factors [7]. Nearly 60% of the reported implantation failures are attributed to endometrial factors [8].

Therefore, to target the most ideal endometrial condition and highest receptivity rates, there should be synchronization between the embryo transfer and implantation window [9]. Enhancing endometrial receptivity has been attempted through hysteroscopic treatment of intrauterine pathologies, myomectomy, or endometrial injury. Additionally, enhancing uterine blood flow through low-dose aspirin and vaginal sildenafil or administration of IVIG also increased endometrial receptivity in patients with RIF [10].

Sequential (two-step) embryo transportation was first proposed by Abramovici et al. [11] as one method for increasing the probability of implantation and reported a clinical pregnancy rate of about 25%. Sequential embryo transfer (SEQET) can increase the availability of embryos at various stages within the implantation window, thus raising the success rate, whereas the clinical pregnancy rate reported with the method of only one embryo transfer was 6.2% [11, 12]. In addition, the embryo itself can stimulate endometrial receptivity [13]. The protocol for SEQET used nowadays was initially proposed by Goto et al. They transplanted a cleavage embryo on the second day, followed by a morula or blastocyst embryo on the fifth day. This resulted in considerably greater pregnancy and implantation rates than the traditional day- 2 transplantation [12].

Despite the use of SEQET in clinical practice nowadays, it remains an area of debate. Some studies have reported the effectiveness of SEQET methods over the conventional day 2 or 3 embryo transfer methods [14, 15], whereas other studies concluded that this method does not offer any advantage over the conventional method [16, 17].

This research seeks to analyze and synthesize clinical information on the influence of SEQET on IVF outcomes and help solve the debate in the current evidence.

## Methods

This study was executed following the “Preferred Reporting Items for Systematic Reviews and Meta-Analyses (PRISMA)” statement [18]. We followed the “Cochrane Handbook guidelines” in doing all the steps [19].

### Literature search and study selection

Two authors have completed the step of literature search through four databases: PubMed, Web of Science, Scopus, and Cochrane. A set of these terms was applied in the formulation of our search strategy (“sequential embryo transfer” and “in vitro fertilization”) and the detailed strategy is shown in Supplementary File 1. Published articles retrieved by the search strategy were collected from inception till May 2024.

The results of the literature search were collected in an Excel sheet and screened through the title and abstract by two independent authors. Then, the retrieved studies were further screened through full text to determine the finally included studies.

### Inclusion criteria

Studies comparing sequential embryo transfer (cleavage followed by blastocyst) to cleavage or blastocyst in women undergoing assisted reproduction either for the first time or repeatedly were conducted. Cleavage was defined as an embryo at day 2 or 3, while blastocyst is an embryo at day 5 or 6. Clinical trials and Cohort studies were included.

### Exclusion criteria

Articles assessing interventions other than sequential embryo transfer were excluded. In addition, case series, case reports, editorials, books, notes, letters to the editors, articles where the full text was not available, and non-English publications were excluded.

### Quality assessment and risk of bias (RoB)

Two investigators separately checked the eligible studies. They evaluated randomized controlled trials (RCTs) using the risk of bias tool 2 (ROB2) from the Cochrane Handbook of Systematic Reviews of Interventions, which includes the following five main domains: “bias arising from the randomization process, bias due to deviations from intended interventions, bias due to missing outcome data, bias in the measurement of the outcome, and bias in the selection of the reported results.” The overall judgments can be low risk of bias, some concern, or high risk of bias [20].

Non-randomized trials were assessed using the ROBINS-I tool from the Cochrane Handbook of Systematic Reviews. The tool consists of seven domains which are “bias due to confounding, bias in selection of participants into the study, bias in classification of interventions, bias due to deviations from intended interventions, bias due to missing data, bias in measurement of outcomes, and bias in selection of the reported result” [21].

The New Castle Ottawa Scale was used to assess the quality of the included cohorts and case–control studies. It has three key domains: “selection, comparability, and outcome.” Thresholds for translating the NOS score were determined according to the Agency for Healthcare Research and Quality (AHRQ): for judgment of good quality: 3 or 4 stars in the selection domain and 1 or 2 stars in the comparability domain and 2 or 3 stars in the outcome/exposure domain; fair quality: 2 stars in the selection domain and 1 or 2 stars in the comparability domain and 2 or 3 stars in the outcome/exposure domain; and the poor quality: 0 or 1 star in the selection domain or 0 stars in the comparability domain or 0 or 1 stars in the outcome/exposure domain [22].

### Data extraction

Data extraction was performed by two investigators separately and revised by a third author. We extracted the general data of each article as (name, publication date, type of study, country, time of realization, inclusion and exclusion criteria, data of the intervention group (SEQET) and control groups (CT or blastocyst transfer), and data about the embryo and RIF). The baseline characteristics of the included patients, such as age, body mass index (BMI), duration of infertility, basal FSH, and mean number of embryos transferred, were extracted.

Moreover, data about clinical pregnancy, chemical pregnancy, ectopic pregnancy, implantation, miscarriage, and multiple pregnancy rates were extracted. Clinical pregnancy is the number of pregnancies confirmed by ultrasound, while chemical pregnancy is the pregnancy diagnosed by beta HCG. The implantation rate was calculated through the detection of sacs on ultrasound.

### Statistical analysis

One author has performed data analysis, and it was revised by another author. It was done by Cochrane Collaboration Review Manager Software (RevMan-computer program version 5.4). The risk ratio (RR) with 95% confidence intervals (CIs) was used to estimate the association strength between SEQET or single-day embryo transfer and different outcomes. The result is significant when the *P*-value is less than 0.05. A fixed effect model was used unless we determined heterogeneity where the random effect model was used in this case. Heterogeneity was defined at a *P*-value less than 0.1. When heterogeneity was detected, an attempt was made to resolve it through the leave-out-one-test. We conducted subgroup analysis according to embryo type (fresh or frozen). Also, subgroups were made according to the study design, either RCTs or non-randomized trials and cohorts. Additionally, we performed a separate analysis for patients with RIF (three or more implantation failure).

## Results

**Fig. 1:**
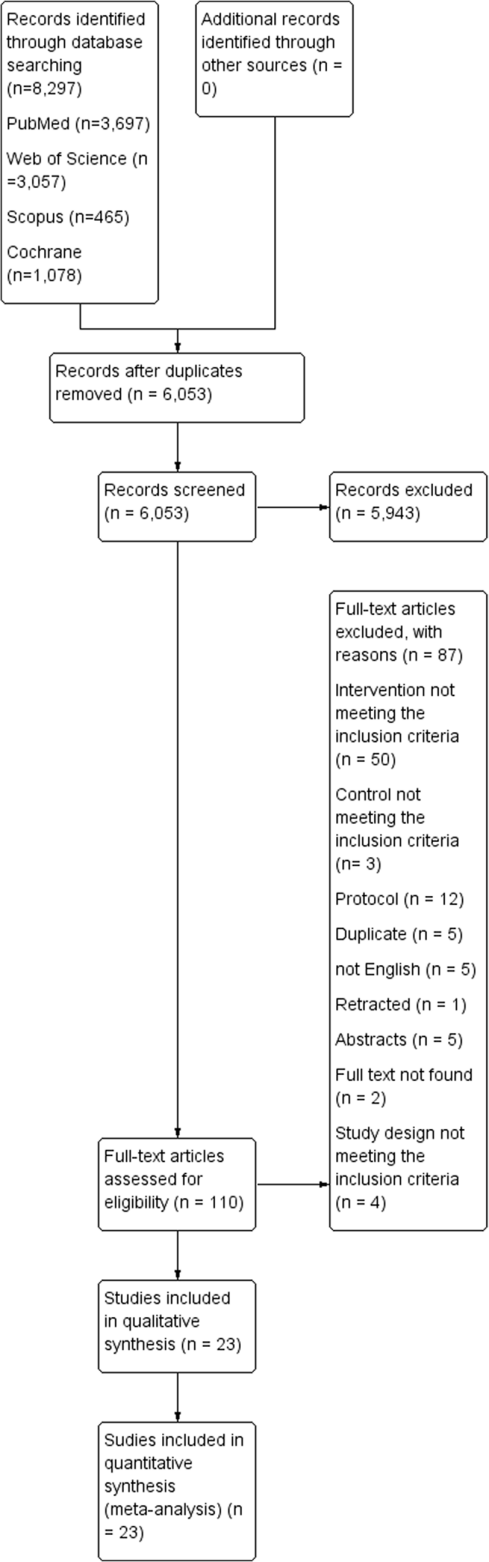
PRISMA flow diagram

The initial literature search through the previously mentioned databases has revealed 8297 articles where 2244 of them were duplicates (Fig. 1). The number of articles identified through each database was as follows: 3697 in PubMed, 1078 in Cochrane, 3057 in WOS, and 465 in Scopus. Title and abstract screening were done to 6053 articles and retrieved 110 articles, which were then screened by the full text. The authors have excluded 87 articles for the following reasons: case reports, case series, editorials, reviews, non-English articles, and duplicates. Eventually, 23 studies were eligible for inclusion in our study [14–17, 23–41]. The study flow diagram is shown in Fig. 1.

Fifteen of the included studies were prospective or retrospective cohorts [14, 17, 24–26, 28–32, 34, 38–41], three were non-randomized trials [16, 23, 35], and five were RCTs [15, 27, 33, 36, 37] (Table 1.). Eleven studies have used fresh embryo transfer [14, 16, 23, 24, 27–29, 33, 35, 37, 38], 11 studies have used frozen embryo transfer [15, 17, 25, 26, 30, 31, 34, 36, 39–41], and both types of embryo transfer were used in one study [32]. RIF, defined as 3 or more implantation failures, was reported in 13 studies[14, 15, 17, 24, 25, 27, 30–33, 36, 37, 41]. Li et al. [30] stratified the patients according to the number of implantation failures into two implantation failures, three implantation failures, and three or more implantation failures. Moreover, Palshetkar et al. [34] defined RIF as two or more implantation failures.

**Table 1.** Summary of the included studies

Author and year	Study design, country, and time of realization	Participants and main inclusion criteria	Exclusion criteria of importance	Intervention	Control	Outcomes	ET (frseh/frozen)	RIF (3 or more implantation failures)
Bongso et al. [23]	Non-randomized clinical trial, Singapore	Women with at least cycle failures, aged from 28 to 41 years	N/A	*N* = 50 women had sequential transfer on day 2 and then day 5/6	*N* = 41 had transfer at D3, *N* = 19 had transfer at D5	Clinical pregnancy rates, implantation rates, multiple pregnancy, miscarriage	Fresh	
Ashkenazi et al. [16]	Non-randomized clinical trial, Israel, between October 1997 and December 1998	Women attending ART with more than five embryos	N/A	*N* = 136 traditional early transfer of embryos on day 2 or 3, and a second (consecutive) transfer of one or more blastocysts on day 5 or 6	*N* = 139 traditional early transfer	Clinical pregnancy, implantation rate, multiple pregnancy	Fresh	
Kyonq et al. [29]	Cohort study, Japan, between January 2001 and December 2002	Women attending ART clinic undergoing IVF/ICSI	N/A	*N* = 137 women who had experienced several ART failures has a two-step (consecutive) transfer	*N* = 460 early embryo transfer, *N* = 88 blastocyst transfer	Implantation rate, pregnancy rate, miscarriage rate, multiple pregnancy rate	Fresh	
Phillips et al. [35]	Non-randomized clinical trial, between January 2001 and July 2002	Women undergoing IVF/ICSI	N/A	*N* = 110 sequential transfers at days 3 and 5/6 transfer	*N* = 32 embryo transfer on day 3	Chemical pregnancy, clinical pregnancy, ectopic pregnancy, miscarriage, ongoing pregnancy, multiple pregnancy	Fresh	
Machtinger et al. [32]	Cohort, Israeli, between March 1999 and May 2004	Women with 3 or more failed IVF cycles	N/A	N = 66 days 3 and 5/6 transfer	*N* = 117 day 3 transfer	Implantation rate, clinical pregnancy rate, multiple pregnancy rate	Both	RIF
Almog et al. [14]	Retrospective cohort study, Israel, between April 2004 and May 2005	Women undergoing IVF with at least five good quality embryos	Women treated for preimplantation genetic diagnosis	*N* = 65 Double transfer on day 2–3 and on day 5	*N* = 66 day 2/3 embryo transfer	Clinical pregnancy rates, multiple pregnancy rate	Fresh	RIF
Fang et al. [24]	Cohort study, China, from August 2010 to December 2011	RIF patients undergoing IVF with good quality embryos	Thrombophilia genetic abnormalities, or endometrial abnormalities	*N* = 66 day- 3 and day- 5 transfer	*N* = 85 day2/3 once-only transfer, *N* = 29 day- 5 once-only transfer	Implantation rate, clinical pregnancy rate, miscarriage, multiple pregnancy rate	Fresh	RIF
Yazbeck et al. [38]	Retrospective cohort study, France, between January 2005 and December 2006	Women with 3 good quality embryos	N/A	*N* = 120 two-step (consecutive) procedure	*N* = 280 traditional cleavage-stage ET (day 2/3)	Clinical pregnancy rate, implantation rate, multiple pregnancy rate, miscarriage rate, live birth rate	Fresh	
Madkour et al. [33]	Randomized clinical trial, United Arab Emirates, between April 2008 and March 2011	Women with RIF with good quality embryos	Endometrial abnormalities, thrombophilia or genetic abnormalities	*N* = 74 sequential transfer (day 3 and day 5)	*N* = 73 conventional transfer (day 3)	Clinical pregnancy, implantation rate, multiple pregnancy rate, early pregnancy loss, ongoing pregnancy rate	Fresh	RIF
Tehraninejad et al. [37]	Randomized clinical trial, Iran, between April 2016 and April 2017	Women with normal endometrium, karyotype, and thrombophilia profile and available 5 embryos	Women with medical diseases, abnormal responders to ovarian tests	*N* = 60 sequential transfer on day 3 and day 5	N = 60 blastocysts transfer on day 5	The primary outcome measures were chemical and clinical pregnancy rate	Fresh	RIF
Kaya et al. [28]	Retrospective cohort study, Turkey, between January 2011 and January 2014	Women aged ≤ 40, having ≥ 3 good quality	Patients with uterine anomaly	*N* = 53 sequential day- 3 and day- 5 embryo transfer	*N* = 135 day 2 or 3 embryo transfer	Clinical pregnancy rate	Fresh	
Arefi et al. [15]	Randomized clinical trial, Iran, between January 2020 and September 2021	Women with RIF 20–39 years, with more than three good-quality frozen embryos, were normal fetus, normal immunological and thrombophilia condition, and normal endometrium	Women with major uterine abnormalities endometriosis, severe male factor infertility, inadequate ovarian reserve, and having medical diseases	*N* = 100 sequential transfer day 3/day 5	*N* = 100 D5 ET	Clinical pregnancy and implantation rates. Number of oocytes retrieved, number of transferred embryos, implantation rate, twin pregnancy, and abortion rate	Frozen	RIF
Ji et al. [17]	A retrospective cohort China, between January 2017 and July 2021	Women aged 40 years younger, with normal hysteroscopy, normal karyotype	Patients with a thin endometrium on the transfer day, repeated miscarriages, severe endometriosis or autoimmune diseases	*N* = 77 sequential transfer	*N* = 80 blastocyst transfer group, *N* = 154 cleavage embryo transfer group	Implantation rate, clinical pregnancy rate, miscarriage, multiple pregnancy rate, ongoing pregnancy	Frozen	RIF
Hu et al. [26]	A retrospective cohort, China, from January 2018 to April 2021	Women with poor ovarian reserve	Having endocrine diseases, hydrosalpinx, adenomyosis, hysteromyoma, uterine abnormalities history of recurrent miscarriage. Patients treated for pre-implantation genetic screening	N = 163 Sequential transfer	*N* = 601 cleavage-stage embryos. *N* = 466 blastocysts	The main pregnancy outcomes measured were the live birth rate and multiple pregnancy rate	Frozen	
Li et al. [30]	Retrospective cohort study, China, from January 2016 to October 2021	Women undergoing FET cycles	Women with uterine abnormality	*N* = 558 sequential transfer	*N* = 1779 day 3 embryo transfer, *N* = 506 blastocyst transfer	Implantation rate (IR), clinical pregnancy rate (CPR), abortion rate (AR), ectopic pregnancy rate (EPR), multiple pregnancy rate (MPR), live birth rate (LBR) and neonatal characteristics	Frozen	RIF
Salehpour et al. [36]	Randomized clinical trial, Iran	Women younger than 40 years old undergoing IVF/ICSI having body mass index under 30 and normal serum follicle-stimulating hormone	Uterine abnormalities, hormonal disruptions, inflammatory and autoimmune diseases, polycystic ovary syndrome and ovarian hyper stimulation syndrome, male factor of chromosomal abnormalities	*N* = 102 RIF sequential transfer	*N* = 100 blastocyst transfer	Implantation rate, chemical pregnancy rate, clinical pregnancy rate, ectopic pregnancy, miscarriage rate, multiple pregnancy rate, ongoing pregnancy	Frozen	RIF
Zhou et al. [40]	A retrospective study, China, from July 1, 2020, to June 30, 2021	Women undergoing FET cycles	Woman was 40 years of age or older; transfer is not performed	*N* = 76 sequential transplantation	*N* = 576 early embryo transfer, *N* = 131 blastocyst transplantation	Clinical pregnancy rate, implantation rate, miscarriages rate, multiple pregnancies	Frozen	
Mostafa et al. [27]	RCT, Egypt, from September 2019 till September 2020	Women aged 20–40 years old, with RIF	Women undergoing ICSI for the first time, BMI > 30 kg/m^2^, uterine abnormalities, genetic abnormalities and abnormal ovarian responders	*N* = 40 sequential transplantation	*N* = 40 embryo transfer at day 3	Chemical pregnancy, clinical pregnancy, ongoing pregnancy	Fresh	RIF
Palshetkar et al. [34]	Retrospective cohort, India, from 1 April 2022 to 31 March 2023	Women undergoing FET cycles	N/A	*N* = 61 sequential embryo transfer	*N* = 262 early embryo transfer at day 3, *N* = 109 embryo transfer at day 5	Clinical pregnancy, ectopic pregnancy	Frozen	
Zou et al. [41]	Retrospective cohort, China, from January 2021 to March 2022	Women aged: 20–40 years who failed to achieve a clinical pregnancy after transfer of at least four good-quality embryos in at least of three fresh or frozen cycles	Uterine abnormalities, adenomyosis, uterine adhesions, contraindications to ART and pregnancy; abnormal genes; severe hydro salpinx and not receiving treatment for fibroids	*N* = 65 sequential embryo transfer	*N* = 72 cleavage embryo transfer, *N* = 29 blastocyst embryo transfer	Implantation rate, clinical pregnancy, ongoing pregnancy, ectopic pregnancy, live birth, early miscarriage, twin pregnancy	Frozen	RIF
Gao et al. [25]	Retrospective cohort, China, from January 2020 to June 2022	Women with three or more implantation failure	Abnormal karyotyping, those using donor oocytes, women with a thin endometrium and women with autoimmune disorders	*N* = 302 sequential embryo transfer	*N* = 797 cleavage-stage embryo transfer, *N* = 493 blastocyst embryo transfer	Implantation rate, clinical pregnancy and multiple pregnancy, chemical pregnancy, miscarriage, and ectopic pregnancy	Frozen	RIF
Li et al. [30]	Retrospective cohort, China, from December 2017 to December 2021	Women undergoing FET using hormone placement therapy; patients with at least two failure cycles	Abnormal karyotyping, abnormalities in thrombosis or immune screening; sever intrauterine adhesions; uterine abnormalities; endometriosis; endometrial tuberculosis	*N* = 360 sequential embryo transfer	*N* = 1080 conventional embryo transfer at day 3 or 5	Clinical pregnancy rate, implantation rate, multiple pregnancy rate, miscarriage rate	Frozen	RIF
Zhao et al. [39]	Retrospective cohort, China, from October 2019 to October 2021	Women aged ≤ 40 with at least 2 consecutive embryo transfer failures who had two embryos transferred in the FET cycle	Transfer cycles with all embryos transferred at the cleavage stage, chromosomal abnormalities, incomplete or missing data, uncontrolled endocrine diseases affecting pregnancy, autoimmune disorders, and endometrial abnormalities	*N* = 69 sequential embryo transfer	*N* = 192 blastocyst transfer	Implantation rate, clinical pregnancy rate, multiple pregnancy rate, miscarriage rate, Live birth rate, premature birth rate, cesarean section rate	Frozen	

*ET* embryo transfer, *RIF* repeated implantation failure, *IVF* in vitro fertilization, *ICSI* intracytoplasmic sperm injection, *ART* assisted reproductive technology, *FET* frozen embryo transfer, *N/A* not available

Most of the included women were in the fourth decade of age (Table 2). The mean duration of infertility was reported by 14 studies, and it ranged from 3.71 ± 2.67 to 10.09 ± 4.92 years in the SEQET group.

**Table 2:** Baseline characteristics of the included population

Study ID	Study groups	Age in years mean (SD)	BMI mean (SD)	Duration of infertility in years mean (SD)	Basal FSH (IU/l) Mean (SD)	No. of embryos transferred mean (SD)
Bongso et al. [23]	Sequential	36.2 (3)	N/A	N/A	N/A	3.8 (0.5)
	D3	35.3 (2.9)	N/A	N/A	N/A	2.4 (0.7)
	D5	34.6 (3.2)	N/A	N/A	N/A	2.5 (0.6)
Ashkenazi et al. [16]	Sequential	31.1 (4.9)	N/A	N/A	N/A	1.9 (0.96)
	D3	32.2 (5.6)	N/A	N/A	N/A	3 (0)
Kyonq et al. [29]	Sequential	35.4 (4.5)	N/A	N/A	N/A	1.65 (0.78)
	D3	35.5 (4.8)	N/A	N/A	N/A	2.5 (1)
	D5	35.5 (4.5)	N/A	N/A	N/A	2.5 (0.9)
Phillips et al. [35]	Sequential	34.7 (3.48)	N/A	N/A	N/A	3 (0.67)
	D3	35 (4.36)	N/A	N/A	N/A	2 (0.75)
Machtinger et al. [32]	Sequential	30. 7(3.2)	N/A	N/A	5.7 (1.8)	3.9 (0.9)
	D3	31 (2.9)	N/A	N/A	5.2 (2.2)	3.9 (1)
Almog et al. [14]	Sequential	34.3 (0.7)	N/A	N/A	N/A	3.7 (0.02)
	D3	34.7 (0.1)	N/A	N/A	N/A	3.6 (0.02)
Fang et al. [24]	Sequential	34.1 (3.2)	N/A	5.7 (3)	5.6 (1.4)	2.4 (1)
	D3	33.9 (4.1)	N/A	5 (2.7)	5.9 (1.3)	2.7 (0.5)
	D5	33.1 (4.5)	N/A	4.8 (3.6)	5.7 (1.7)	1.9 (1.1)
Yazbeck et al. [38]	Sequential	33.07 (4.13)	N/A	N/A	6.08 (1.9)	2.18 (0.41)
	D2/3	33.59 (3.98)	N/A	N/A	6.55 (2.03)	2.24 (0.6)
Madkour et al. [33]	Sequential	34.4 (1.4)	23.47(7.92)	8.3 (0.72)	6.56 (2.31)	N/A
	D3	34 (1.5)	21.98(5.86)	8.54 (0.61)	6.17 (3.08)	N/A
Tehraninejad et al. [37]	Sequential	35.03 (4.35)	N/A	10.09 (4.92)	7.02 (3)	6.73 (1.6)
	D5	34.09 (4.2)	N/A	9.16 (4.93)	7.22 (2.95)	6.28 (1.4)
Kaya et al. [28]	Sequential	29.57 (5.87)	24.6 (4)	7 (5.33)	7 (1.52)	N/A
	D2/3	31.33 (5.25)	24.4 (4.2)	5 (3)	7.07 (2.17)	N/A
Arefi et al. [15]	Sequential	35.06 (4.33)	25.95 (3.75)	6.92 (2.4)	6.23 (1.68)	3.34 (0.699)
	D5	33.9 (4)	25.83 (3.01)	7.48 (2.13)	6.28 (1.82)	3.16 (0.801)
Ji et al. [17]	Sequential	33.39 (3.89)	21.38 (2.72)	4.09 (3.19)	N/A	N/A
	D3	33.63 (3.97)	21.53 (2.61)	3.84 (2.83)	N/A	N/A
	D5	32.5 (3.79)	21.16 (2.65)	3.53 (2.6)	N/A	N/A
Hu et al. [26]	Sequential	35.63 (5.26)	22.03(2.64)	N/A	10.44 (4.59)	2 (1)
	D3	36.07 (5.5)	22.15 (2.95)	N/A	10.44 (4.98)	2 (1)
	D5	35.08 (4.51)	22.04 (3.01)	N/A	9.86 (4.33)	2 (1)
Li et al. [30]	Sequential	33.8 (4.7)	21.5 (2.5)	4.1 (3.2)	N/A	N/A
	D3	35.5 (5.2)	21.6 (2.5)	4.4 (3.3)	N/A	N/A
	D5	33.73 (4.5)	21.6 (2.64)	4 (3.03)	N/A	N/A
Salehpour et al. [36]	Sequential	33.92 (0.4794)	26.93 (0.2366)	7.049 (0.3634)	5.967 (0.2074)	2 (1)
	D5	34.9 (0.5192)	26.47 (0.235)	8.03 (0.3465)	6.457 (0.2169)	2 (1)
Zhou et al. [40]	Sequential	33.37 (4.59)	N/A	3.81 (3.23)	N/A	2.35 (0.48)
	D3	34.06 (5.7)	N/A	3.28 (2.73)	N/A	1.88 (0.34)
	D5	33.69 (5.17)	N/A	3.28 (2.48)	N/A	1.53 (0.52)
Mostafa et al. [27]	Sequential	33.65 (1.69)	25.63 (2.19)	7.26 (0.79)	7.41 (0.91)	3.98 (0.86)
	D3	32.95 (2.4)	26.48 (2.04)	6.92 (1.07)	7.36 (0.93)	3.9 (1.1)
Palshetkar et al. [34]	Sequential	33.71 (5.31)	N/A	N/A	N/A	N/A
	D3	31.76 (5.5)	N/A	N/A	N/A	N/A
	D5	33.99 (6.64)	N/A	N/A	N/A	N/A
Zou et al. [41]	Sequential	31.43 (4.05)	22.11 (2.75)	4.33 (3.79)	N/A	N/A
	D3	32.54 (3.43)	22.7 (3.36)	5.7 (3)	N/A	N/A
	D5	30.9 (3.09)	21.38 (3.1)	5 (3)	N/A	N/A
Gao et al. [25]	Sequential	34.05 (4.51)	22.39 (3.48)	5.52 (3.27)	N/A	1.89 (0.32)
	D3	33.63 (4.27)	22.37 (3.45)	4.91 (3.29)	N/A	2 (0)
	D5	33.67 (4.01)	22.36 (3.23)	5.73 (3.46)	N/A	2 (0)
Li et al. [30]	Sequential	31.45 (4.32)	22.62 (3.2)	3.71 (2.67)	6.65 (2.21)	N/A
	Conventional (D3 and D5/D6)	31.73 (4.47)	22.52 (3.53)	3.73 (2.8)	6.68 (3.75)	N/A
Zhao et al. [39]	Sequential	31.46 (3.15)	21.2 (2.25)	3.98 (2.33)	6.47 (2.81)	N/A
	D5/D6	30.6 (3.49)	21.49 (3.17)	4.07 (2.28)	6.08 (2.08)	N/A

*BMI* Body mass index, *FSH* follicular stimulating hormone, *NA* not available

### Quality assessment

**Fig. 2:**
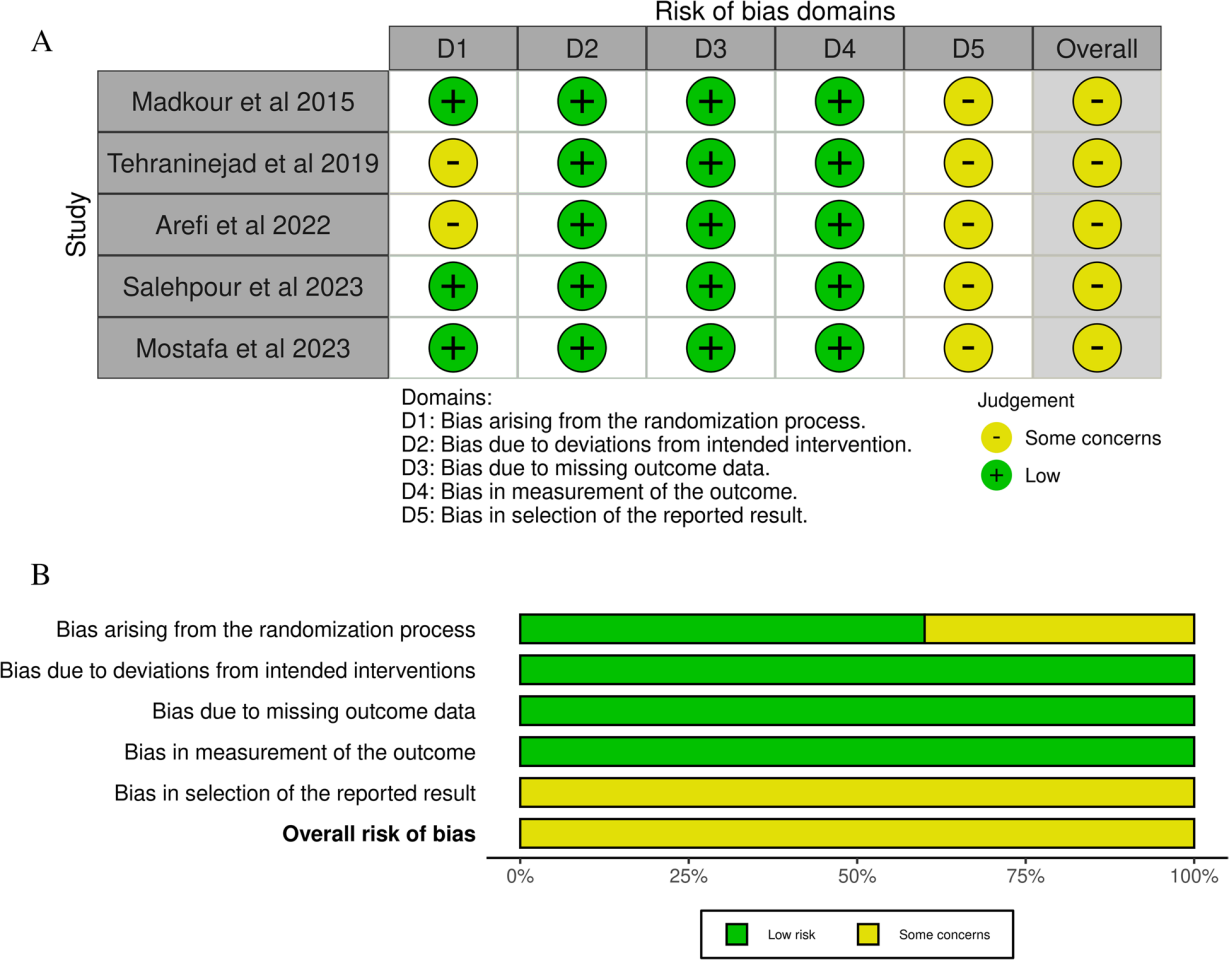
**A** Risk of bias summary. **B** Risk of bias graph

All of the included RCTs have shown some concerns in the overall risk of bias. This was mainly attributed to some concerns in reporting the results. In addition, Tehraninejad et al. [37] and Arefi et al. [15] showed some concerns in the selection domain. The risk of bias summary and graph are shown in Fig. 2A and B.

Regarding the included cohorts, a summary of quality assessment by NOS is shown in Table 3. All of the studies have shown good quality except Kyonq et al. [29]. Kyonq et al. has shown fair quality due to scoring 2 stars in the outcome domain as a result of the inadequacy of follow-up and short follow-up time in addition to scoring one star in the comparability domain.

**Table 3:** Summary of quality assessment of the included cohorts according to NOS

Study	Selection				Comparability	Outcome				Quality
	Representativeness of the exposed cohort	Selection of the non-exposed cohort	Ascertainment of exposure 5	Outcome was not present at start of study	Control for 2 important factors	Assessment of outcome	Follow-up long enough	Adequacy of follow-up of cohort		
Kyonq et al. [29]		*	*	*	*	*				Poor quality
Machtinger et al. [32]		*	*	*	*	*			*	Good quality
Almog et al. [14]		*	*	*	*	*	*		*	Good quality
Yazbeck et al. [38]		*	*	*	*	*			*	Good quality
Fang et al. [24]		*	*	*	*	*			*	Good quality
Kaya et al. [28]		*	*	*	**	*			*	Good quality
Ji et al. [17]		*	*	*	**	*			*	Good quality
Hu et al. [26]		*	*	*	**	*	*		*	Good quality
Li et al. [30]		*	*	*	**	*	*		*	Good quality
Zhou et al. [40]		*	*	*	*	*			*	Good quality
Palshetkar et al. [34]		*	*	*	*	*			*	Good quality
Li et al. [30]		*	*	*	*	*			*	Good quality
Gao et al. [25]		*	*	*	*	*			*	Good quality
Zou et al. [41]		*	*	*	*	*	*		*	Good quality
Zhao et al. [39]		*	*	*	**	*	*		*	Good quality

Regarding the non-randomized trials, they were all associated with a low risk of bias in all of the tool domains, as well as a low overall risk of bias [16, 23, 35] (Table 4).

**Table 4:** Quality assessment of the non-randomized trials according to ROBINS-I

Study domain	Bongso et al. [23]	Ashkenazi et al. [16]	Phillips et al. [35]
Bias due to confounding	Low risk	Low risk	Low risk
Bias in selection of participants into the study	Low risk	Low risk	Low risk
Bias in classification of interventions	Low risk	Low risk	Low risk
Bias due to deviations from intended interventions	Low risk	Low risk	Low risk
Bias due to missing data	Low risk	Low risk	Low risk
Bias in measurement of outcomes	Low risk	Low risk	Low risk
Bias in selection of the reported result	Low risk	Low risk	Low risk
Overall risk of bias	Low risk	Low risk	Low risk

## Outcomes

### SEQET (cleavage transfer followed by blastocyst transfer) vs cleavage embryo transfer CET in all patients

## Clinical pregnancy

**Fig. 3:**
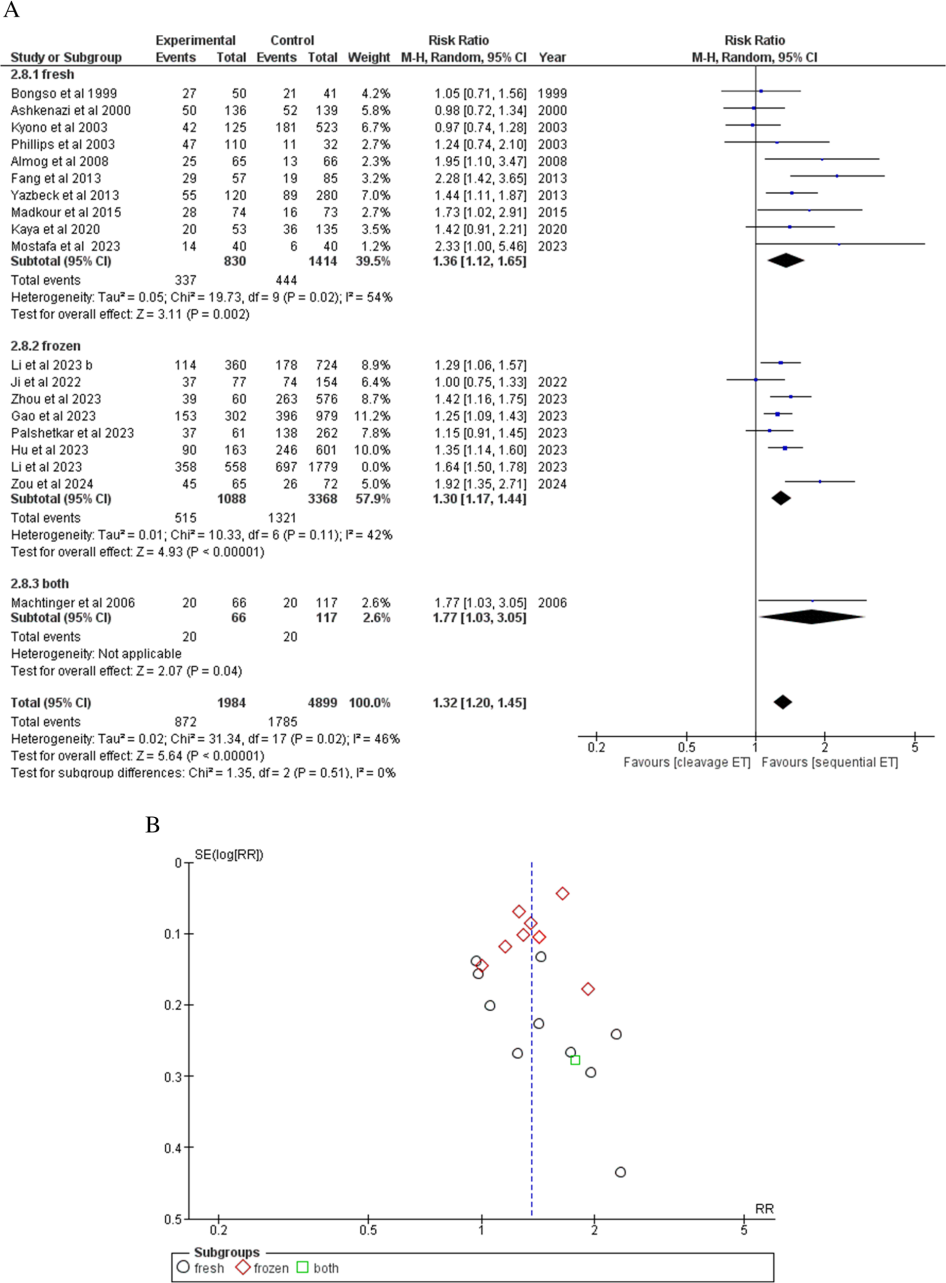
**A** forest plot comparing clinical pregnancy rates. **B** Funnel plot of clinical pregnancy rates

Clinical pregnancy was reported by 19/23 studies. The overall count of patients was 1984 in the SEQET group and 4899 in the CET group. Ten studies have used fresh embryos, eight studies have used frozen embryos, and one study has used both types of embryos. Overall, SEQET has demonstrated a markedly greater clinical pregnancy rate (43.95%) relative to CET (36.45%) under the random effect model (RR = 1.32, 95% CI [1.20, 1.45], *P* < 0.00001) (Fig. 3A). Subgroup differences showed non-significance in the statistical results (*P* = 0.51). The combined studies demonstrated heterogeneity (*P* = 0.02) even the leave-one-out test and subgroup analysis. In the frozen embryos subgroup, heterogeneity was addressed following the exclusion of Li et al. (*P* = 0.1). The analysis showed significant publication bias among the papers included (Fig. 3B).

## Chemical pregnancy

Chemical pregnancy was reported by 6/23 studies, where 4 studies used fresh embryos and 2 studies used frozen embryos. The overall count of patients was 754 in the SEQET group and 1791 in the CET group. SEQET demonstrated markedly improved chemical pregnancy (54.51%) relative to CET (45.17%) under the fixed model (RR = 1.32, 95% CI [1.21, 1.44], *P* < 0.0001) (Figure S1). Subgroup differences showed non-significance in the statistical results (*P* = 0.06). Homogeneity was detected among the entire studies (*P* = 0.44).

## Ectopic pregnancy

Ectopic pregnancy was reported by 6/23 studies. The overall count of patients was 730 in the SEQET group and 1514 in the CET group. No advantage was exhibited by one of the groups over the other, under the fixed model (RR = 0.62, 95% CI [0.33, 1.15], *P* = 0.13) (Figure S2). The combined studies were homogeneous (*P* = 0.32).

## Implantation rate

The implantation rate was reported by 15 studies. Seven studies have used fresh embryos, seven studies have used frozen embryos, and one study has used both types of embryos. Overall, SEQET has markedly demonstrated a greater implantation rate (27.2%) relative to the CET group (21.58%) under the random model (RR = 1.30, 95% CI [1.10, 1.53], *P* = 0.002) (Figure S3a). However, in the subgroup of fresh embryos, SEQET did not show any advantage over CT (RR = 1.11, 95% CI [0.79, 1.57], *P* = 0.54).

A significant subgroup difference was detected between the study subgroups favoring the frozen embryo transfer group (*P* = 0.02). The combined studies were heterogeneous (*P* < 0.00001) and heterogeneity was not solved by the leave-one-out test or subgroup analysis. The analysis showed significant publication bias among the papers included (Figure S3b).

## Live birth

Live birth was reported by 4/23 studies. The overall count of patients was 906 in the SEQET group and 2732 in the CET group. SEQET demonstrated an improved live birth number relative to the CET group, under the random effect model (RR = 1.76, 95% CI [1.38, 2.24], *P* < 0.0001) (Figure S4). The combined studies were heterogeneous (*P* = 0.006).

## Miscarriage

The miscarriage rate was reported by 12/23 studies. The overall count of patients was 1037 in the SEQET group and 2196 in the CET group. Five studies have used fresh embryos, and seven studies have used frozen embryos. Overall, statistical analysis confirmed that no group was superior to the other under the fixed effect model (RR = 1.03, 95% CI [0.87, 1.23], *P* = 0.72) (Figure S5a). The analysis also demonstrated non-significance in the subgroup difference results (*P* = 0.23). The combined studies were homogenous (*P* = 0.11). The analysis showed publication bias among the included studies (Figure S5b).

## Multiple pregnancies

Multiple pregnancies were reported by 16/23 studies. The overall count of patients was 1159 in the SEQET group and 2302 in the CET group. Eight studies have used fresh embryos, six studies have used frozen embryos, and only one study has used both types of embryos. Statistical analysis confirmed that neither of the groups was superior to the other, under the fixed effect model (RR = 0.99, 95% CI [0.88, 1.11], *P* = 0.85) (Figure S6a).

The subgroup differences showed significance in the results (0.04). The pooled studies have demonstrated homogeneity (*P* = 0.16). The analysis showed no serious publication bias among the papers included (Figure S6b).

### SEQET (experimental group) vs CET (control group) in patients with RIF

## Clinical pregnancy

Clinical pregnancy among patients with RIF was reported in 9/23 studies. The overall count of patients was 1304 in the SEQET group and 3365 in the CET group. Four studies have used fresh embryos, four studies have used frozen embryos, and only one study has used both types of embryos.

**Fig. 4:**
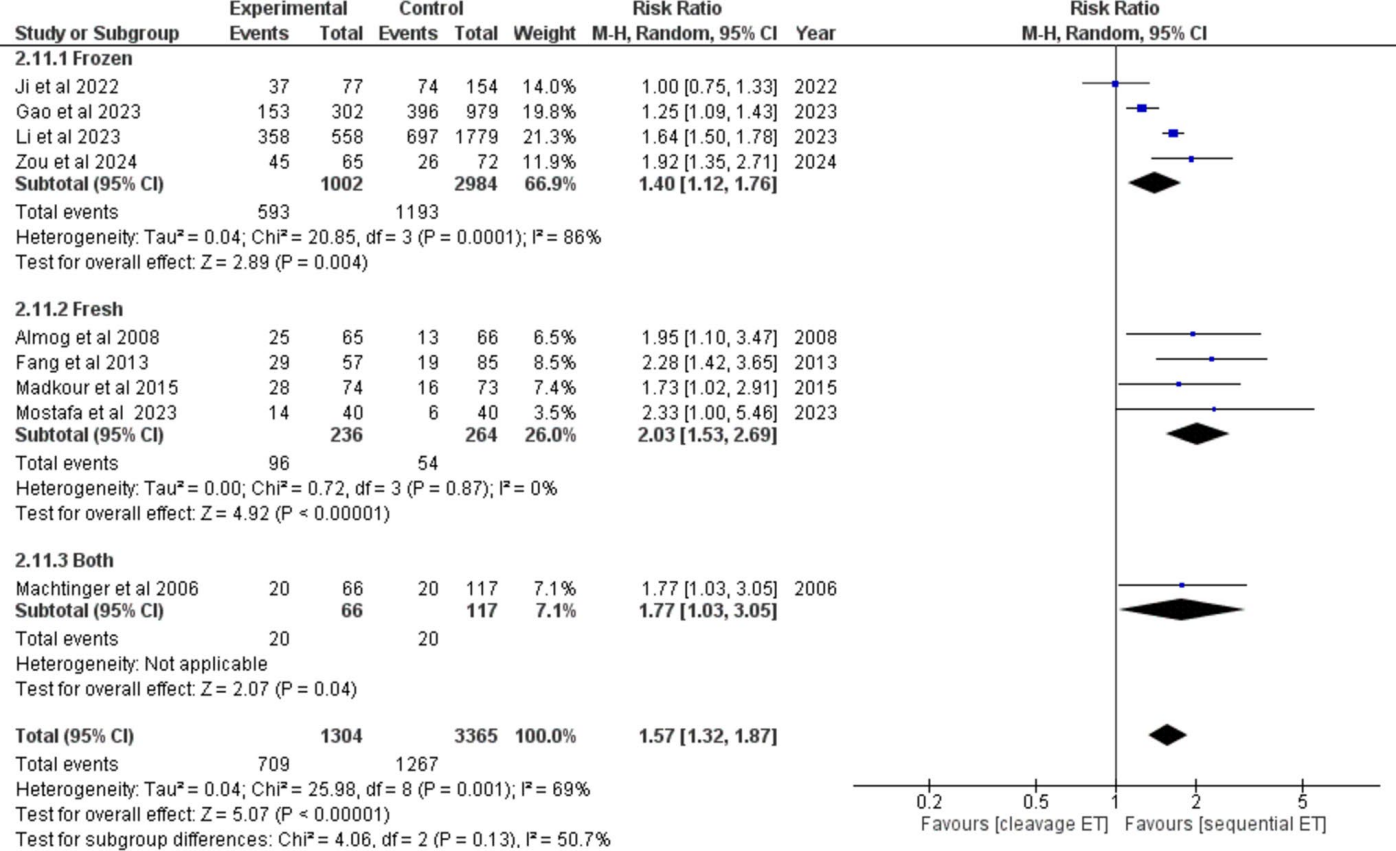
Forest plot comparing clinical pregnancy in patients with RIF

SEQET has demonstrated a markedly improved clinical pregnancy rate relative to CET, under a random model (RR = 1.57, 95% CI [1.32, 1.87], *P* < 0.00001) (Fig. 4). Subgroup differences showed non-significance in the results (*P* = 0.13). The combined studies were heterogeneous (*P* = 0.001). However, studies in the fresh subgroup were homogeneous (*P* = 0.87).

## Miscarriage

Miscarriages among patients with RIF were reported by 6/23 studies. The overall count of patients was 650 in the SEQET group and 1228 in the CET group. Two studies have used fresh embryos and four studies have used frozen embryos. Analysis confirmed that neither of the groups was superior to the other, under the random effect model (RR = 0.92, 95% CI [0.73, 1.17], *P* = 0.50) (Figure S7). Subgroup differences demonstrated non-significance in the results (*P* = 0.47). The heterogeneity among the included studies was solved by the leave-one-out test (*P* = 0.5).

## Implantation rates

Implantation rates among patients with RIF were reported by 7/23 studies. Two studies have used fresh embryos, four studies have used frozen embryos, and only one study has used both. SEQET markedly demonstrated an improvement in implantation relative to CET, under the random model (RR = 1.67, 95% CI [1.34, 2.09], *P* < 0.00001) (Figure S8).

Subgroup differences demonstrated non-significance in the results (*P* = 0.13). Collectively, the studies showed heterogeneity (*P* = 0.0007). However, studies in the subgroup of fresh embryo transfer were homogeneous (*P* = 0.23).

## Multiple pregnancies

Multiple pregnancies among patients with RIF were reported by 8/23 studies. The overall count of patients was 695 in the SEQET group and 1261 in the CET group. Two studies have used fresh embryos, four studies have used frozen embryos, and only one study has used both. Analysis confirmed that neither of the groups was superior to the other, under a fixed model (RR = 1.02, 95% CI [0.87, 1.20], *P* = 0.79) (Figure S9). The analysis demonstrated significance in the subgroup differences (*P* = 0.05). Collectively, the studies showed homogeneity (*P* = 0.12).

### SEQET (experimental group) vs blastocyst transfer (control group) in all patients

## Clinical pregnancy

**Fig. 5:**
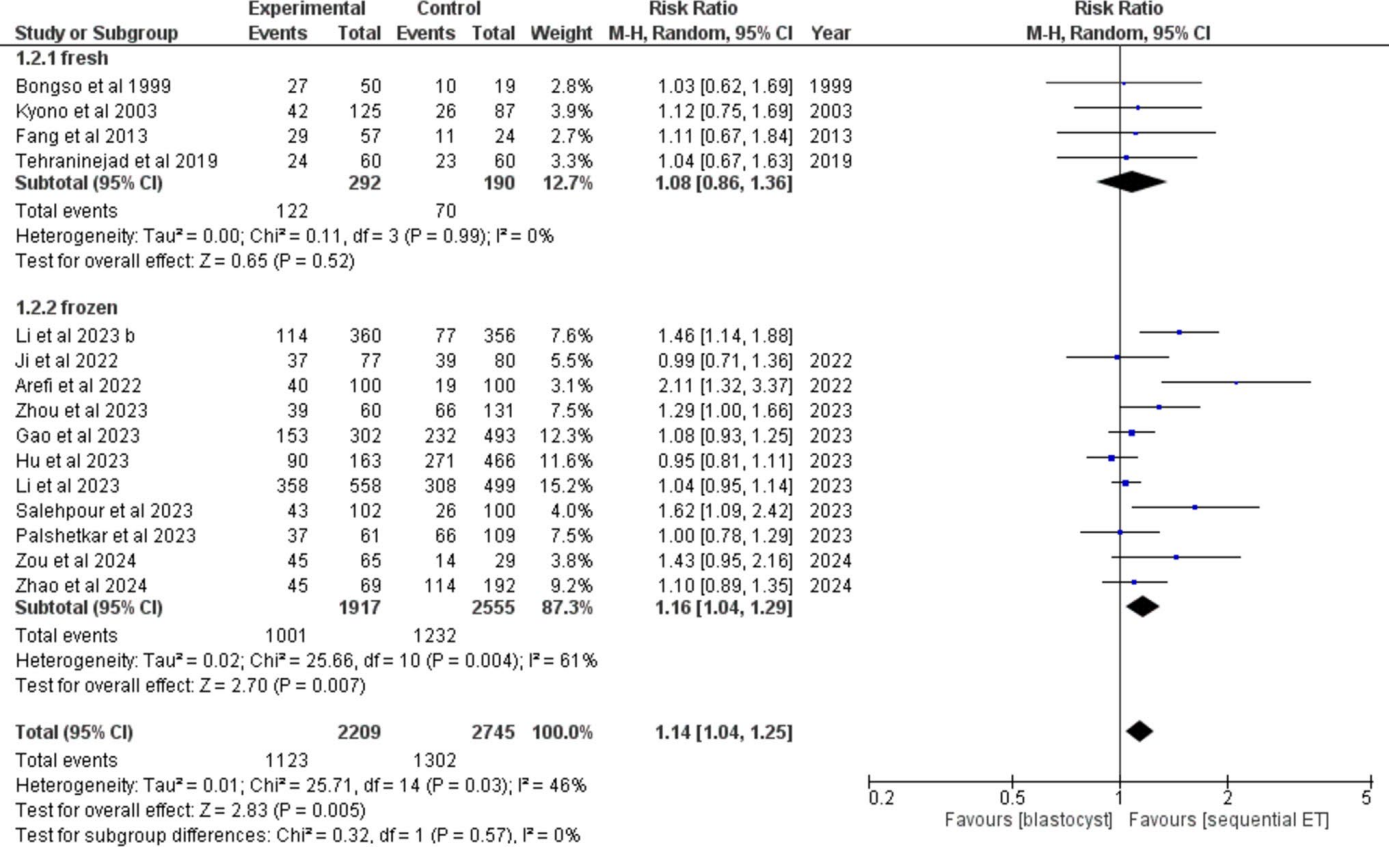
Forest plot comparing clinical pregnancy in sequential ET vs blastocyst

Clinical pregnancy was reported by 15/23 studies. Four studies have used fresh embryos, and eleven studies have used frozen embryos. The overall count of patients was 2209 in the SEQET group and 2745 in the blastocyst group. Overall, the clinical pregnancy demonstrated marked improvement among those with SEQET (50.84) relative to blastocyst transfer (47.43%), under the random model (RR = 1.14, 95% CI [1.04, 1.25], *P* = 0. 003) (Fig. 5).

Subgroup differences showed non-significance in the results (*P* = 0.57). The pooled studies were heterogeneous (*P* = 0.03), but the fresh embryos subgroup showed homogeneity among the studies (*P* = 0.004).

Subgroup analysis was also made based on the study design. Three RCTs and 12 non-randomized trials and cohorts reported the outcome. Overall, the results were significant and favored the sequential embryo transfer group (RR = 1.14, 95% CI [1.04, 1.25], *P* = 0.006) (Suppl. Figure 10). There was a significant difference between the subgroups (*P* = 0.001). Collectively, the studies demonstrated heterogeneity (P = 0.02). Whereas studies in the non-randomized and cohort’s subgroup and RCTs subgroup were homogeneous (*P* = 0.41 and 0.24, respectively), after the leave-one-out test. The analysis detected publication bias among the papers included (Figure S11).

## Chemical pregnancy

Chemical pregnancy was reported by 4/23 studies. The overall count of patients was 627 in the SEQET and 1119 in the blastocyst group. The analysis confirmed that neither of the groups was superior to the other, under the fixed effect model (RR = 1.01, 95% CI [0.92, 1.10], *P* = 0.88) (Figure S12). The pooled studies were homogeneous (*P* = 0.16).

## Ectopic pregnancy

Ectopic pregnancy was reported by 6/23 studies. The overall count of patients was 726 in the SEQET group and 917 in the blastocyst group. The analysis confirmed that neither of the groups was superior to the other, under the fixed effect model (RR = 1.39, 95% CI [0.66, 2.94], *P* = 0.38) (Figure S13). Collectively, the studies were homogenous (*P* = 0.38).

## Implantation rates

Implantation was reported by 13/23 studies. Three studies have used fresh embryos, and ten studies have used frozen embryos. Analysis confirmed that neither of the groups was superior to the other, under a random model (RR = 1.06, 95% CI [0.90, 1.25], *P* = 0.49) (Figure S14a). Subgroup differences demonstrated significance in the results (0.02). Collectively, the studies showed heterogeneity (*P* < 0.00001). However, studies of the fresh embryos subgroup were homogenous (*P* = 0.31). Significant publication bias was detected among the studies (Figure S14b).

## Miscarriages

Miscarriages were reported by 12/23 studies. Two studies have used fresh embryos and ten studies have used frozen embryos. The overall count of patients was 1035 in the SEQET group and 1203 in the blastocyst group. The analysis confirmed that neither of the groups was superior to the other, under a fixed model (RR = 0.95, 95% CI [0.79, 1.13], *P* = 0.54) (Figure S15). Subgroup differences demonstrated non-significance in the results (*P* = 1). Collectively, the studies showed homogeneity (*P* = 0.33).

## Multiple pregnancies

Multiple pregnancies were reported by 14/23 studies. Four studies have used fresh embryos, and ten studies have used frozen embryos. The analysis confirmed that neither of the groups was superior to the other, under the random effect model (RR = 0.84, 95% CI [0.96, 1.01], *P* = 0.07) (Figure S16a). Subgroup differences demonstrated non-significance in the results (*P* = 0.96). Collectively, the studies demonstrated homogeneity (*P* = 0.11) after excluding Bongso et al. [23] from the fresh subgroup. Analysis showed no significant publication bias in multiple pregnancies (Figure S16b).

## Live births

Live birth was reported by 4/23 studies. The overall count of patients was 885 in the SEQET group and 1186 in the blastocyst group. The analysis confirmed that neither of the groups was superior to the other, under the fixed effect model (RR = 1.03, 95% CI [0.94, 1.13], *P* = 0.53) (Figure S17). Collectively, the studies demonstrated homogeneity (*P* = 0.13).

### SEQET (experimental group) vs blastocyst transfer (control group) in patients with RIF

## Clinical pregnancy rate

**Fig. 6:**
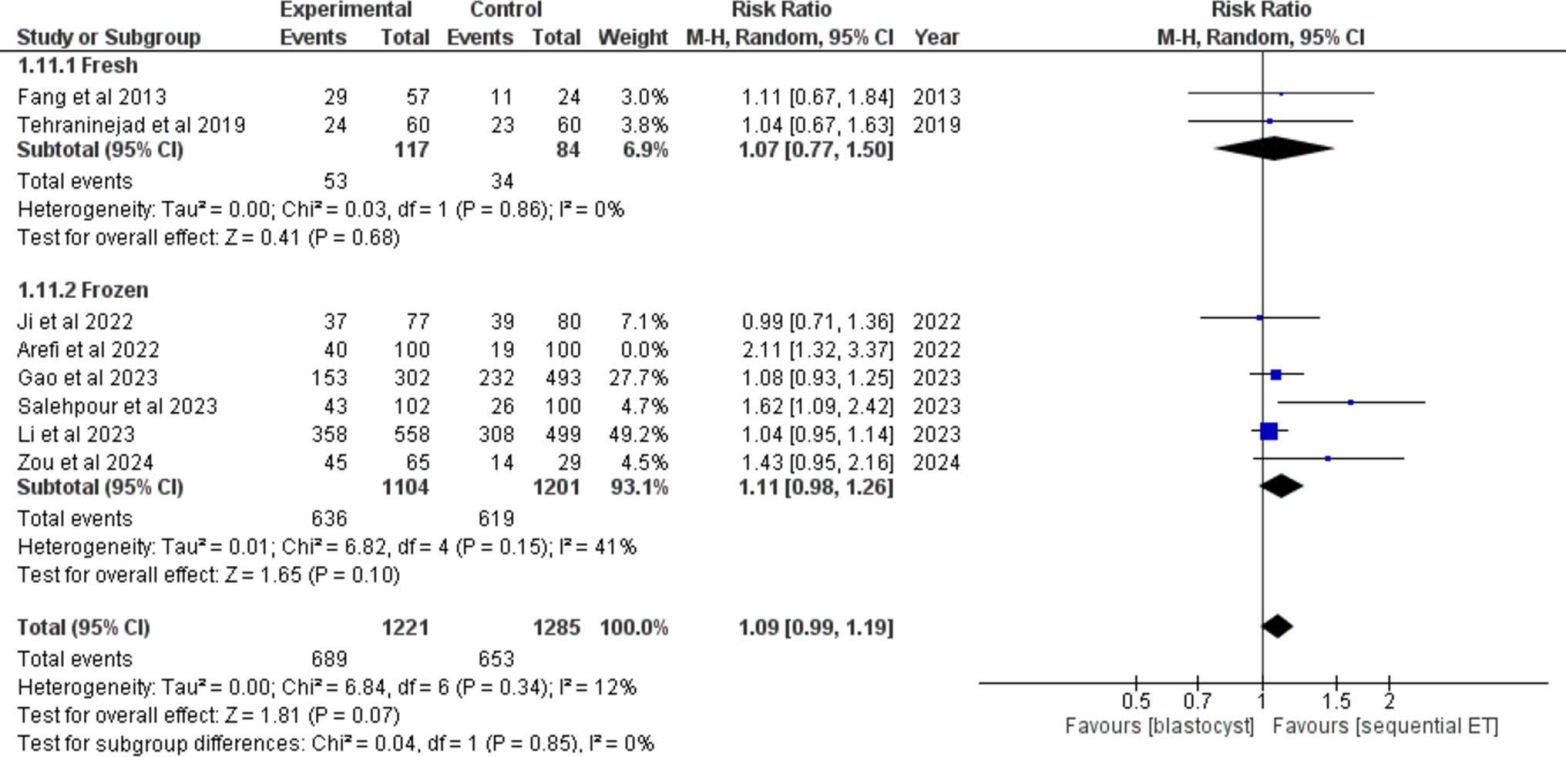
Forest plot comparing clinical pregnancy rate between sequential ET and blastocyst transfer in patients with RIF

The clinical pregnancy rate was reported by 8/23 studies. Two studies have used fresh embryos, and six studies have used frozen embryos. The overall count of patients was 1221 in the SEQET group and 1285 in the blastocyst group. SEQET demonstrated no significant difference in clinical pregnancy under a random model (RR = 1.09, 95% CI [0.99, 1.19], *P* = 0.07) (Fig. 6). Subgroup differences demonstrated non-significance in the results (*P* = 0.85). Collectively, the studies were homogeneous after the exclusion of Arefi et al. (*P* = 0.15).

## Implantation rate

The implantation rate was reported by 7/23 studies. Analysis confirmed that neither of the groups was superior to the other, under a random model (RR = 1.18, 95% CI [0.90, 1.55], *P* = 0.23) (Figure S18). Collectively, the studies were heterogeneous (P < 0.00001).

## Miscarriage

Miscarriages were reported by 7/23 studies. Analysis confirmed that neither of the groups was superior to the other, under a random model (RR = 1.17, 95% CI [0.81, 1.68], *P* = 0.40) (Figure S19). The pooled studies were homogeneous after leaving Gao et al. (*P* = 0.50).

## Multiple pregnancies

Multiple pregnancies were reported in 8/23 studies. Two studies have used fresh embryos, and six studies have used frozen embryos. The overall count was 729 in the SEQET group and 672 in the blastocyst group. SEQET demonstrated marked improvement in the multiple pregnancies rate under a fixed model (RR = 0.75, 95% CI [0.65, 0.87], *P* < 0.0001) (Figure S19). Subgroup differences demonstrated non-significance in the results (*P* = 0.99). Collectively, the studies showed homogeneity (*P* = 0.14).

## Discussion

This article compared the outcomes of sequential embryo transfer to the outcomes of cleavage transfer and the outcomes of blastocyst transfer separately. We found that SEQET demonstrated a marked increase in clinical and chemical pregnancy rates, implantation rates, and live births in comparison to the CET, whereas sequential ET did not show a significant advantage over cleavage transfer in ectopic pregnancy, miscarriage, multiple pregnancy, and implantation rates with the fresh embryos group.

In agreement with our results, Teng et al. found that SEQET markedly demonstrated greater chemical and clinical pregnancy rates when compared to cleavage transfer. Furthermore, in our study, we found that SEQET improved the live birth rate. On the other hand, there was a difference regarding the implantation rate where our analysis significantly favored SEQET, while Teng et al. [42] denied any advantage for one of the groups over the other regarding the implantation rate.

Moreover, in our analysis, we compared the studies that used fresh embryos to those that used frozen embryos. The implantation rates were significantly higher and favored the SEQET group over CET among studies that used frozen embryos. Conversely, the results were comparable between the groups in studies using fresh embryos. Likewise, a systematic review conducted by Zhang et al. has reported a significant increase in the clinical and chemical pregnancy outcomes that favored SEQET over CET. Their results in the implantation rates also contradict our results where they did not find a significant difference between SEQET and CET [43].

In comparing SEQET to blastocyst transfer, we did not find any significant difference among the groups in all the examined outcomes except for the clinical pregnancy rate. The clinical pregnancy rate was found to be significantly higher in subgroups based on the study design. Teng et al. also compared SEQET to blastocyst transfer. They did not find a significant difference between the groups except for the higher clinical pregnancy rates among the SEQET, which came in agreement with our results [42]. On the other hand, Zhang et al. [43] denied any advantage for one of the groups over the other in all of the reported outcomes.

A separate analysis was made for patients with RIF. SEQET showed significantly higher rates in clinical pregnancy and implantation rates in comparison with CET. However, no significant differences were found in miscarriage and multiple pregnancy rates. Likewise, Zhang et al. [43] have found a significant increase in clinical pregnancy rates among the SEQET group, but no significant difference was detected in miscarriage and multiple pregnancy rates. Moreover, in our analysis, SEQET has significantly increased clinical pregnancy and reduced multiple pregnancy rates over blastocyst transfer.

Teng et al. have also made a subgroup analysis for the RIF group, but the control group included both the cleavage and blastocyst transfer. They reported significant improvements in the chemical and clinical pregnancy rates that favored SEQET. On the other hand, they did not report significant differences in implantation rates or miscarriage rates, which is consistent with our results [42].

Our results support the hypothesis that the higher clinical pregnancy rates in the SEQET group may be attributed to the second blastocyst transfer. Blastocysts have considerable growth potential, and extended in vitro culture reduces the risk of transferring embryos with defective chromosomes. Blastocyst transfer improves endometrial synchrony and receptivity, leading to increased implantation rates [44]. Glujovsky et al.’s systematic review and meta-analysis has compared the outcomes of blastocyst transfer to those of CET. They concluded that blastocyst transfer is associated with higher pregnancy rates than cleavage transfer which agrees with the previously mentioned hypothesis [45].

A possible explanation is that exposing early-stage embryos to the environment of the uterus, especially those with high amounts of estrogen from superovulation, is physiologically premature [46]. Embryos pass through the fallopian tubes and do not reach the uterus until the morula stage, a process that requires at least day 4 of in vitro preparation [47]. Another explanation postulates that the uterus provides different environmental conditions than those of the ovaries and the tubes. Consequently, this may be stressful for the embryo to survive and implant if transferred at the embryo stage [48]. Moreover, it was reported in previous studies that blastocyst transfer has shown lower rates of chromosomal abnormalities and a better chance of giving a good-quality embryo [49, 50]. SEQET comprises two transfer techniques. Previously transmitted cleavage embryos activate an unknown adhesion factor, leading to an increased immune response. Additionally, the previously transferred embryo cultures with the endometrial environment make the endometrium more receptive to the second transfer [44].

The main strength of our study is that we made subgroups for the fresh embryos and frozen embryos and separately analyzed their data. Moreover, we investigated the efficacy of SEQET in improving live births. This outcome was not investigated in the previous meta-analyses [42, 43]. In addition, the analysis of patients with RIF outcomes was done for SEQET in comparison to CET and blastocyst embryos separately. Moreover, most of the included studies are of high quality which enables the generalizability of our results and the possibility of having a reliable conclusion. The limitations of our study are that a small number of the included studies reported live births, miscarriages, and ectopic pregnancies. Thus, we recommend for the upcoming studies to prolong the follow-up period and investigate the rates of live births, miscarriages, and ectopic pregnancies.

## Conclusion

This research discovered that sequential embryo transfer significantly enhanced clinical and chemical pregnancy, as well as implantation. The transfer of frozen embryos improved implantation with SEQET relative to CET. Also, frozen embryos contributed to a higher clinical pregnancy rate with SEQET when compared to blastocyst transfer. These findings should be interpreted cautiously due to the heterogeneity of the studies included.

## Supplementary Information

Below is the link to the electronic supplementary material. 

ESM1 (DOCX 381 KB)

## Data Availability

All data generated or analyzed during this study are included in this published article or in the data repositories listed in References.

## Code availability

N/A.
