# Supplementary material for: Sequential embryo transfer efficacy in enhancing pregnancy outcomes: a systematic review and meta-analysis
Source: J Assist Reprod Genet. 2025 May 21;42(7):2119–40. doi: 10.1007/s10815-025-03487-5 (PMC12356793; doi:10.1007/s10815-025-03487-5)
Supplement: Supplementary file 1 — (DOCX 381 KB) [file 10815_2025_3487_MOESM1_ESM.docx]

**Sequential embryo transfer in women undergoing assisted reproduction: a systematic review and meta-analysis**

**Search strategy**

Search date : 5/ 20224

**PubMed:**

(sequential transfer OR sequential embryo transfer OR consecutive embryo transfer OR sequential embryo transplantation OR sequencing embryo transfer OR interval double transfer OR two-step transfer OR two-step embryo transfer OR Two-step consecutive transfer OR double embryo transfer OR sequential transplantation) AND ("Fertilization in Vitro"[Mesh] OR In Vitro Fertilization OR IVF OR In Vitro Fertilizations OR Test-Tube Fertilization OR Test-Tube Fertilizations OR Test Tube Fertilization OR Fertilizations in Vitro OR Test-Tube Babies OR Test Tube Babies OR Test-Tube Baby OR Recurrent in Vitro Fertilization Failure OR Recurrent in-vitro Fertilization Failure OR Recurrent IVF failure OR Recurrent Failure of in vitro Fertilization OR "Sperm Injections, Intracytoplasmic"[Mesh] OR Intracytoplasmic Sperm Injection OR Intracytoplasmic Sperm Injections OR ICSI OR Embryo Implantation OR Poor Implantation OR Implantation Failure OR Repeated Implantation Failure OR Recurrent Implantation Failure OR Recurrent Failure of Implantation OR Recurrent Failure to Implant OR Repeat Failure To Implant OR Recurrent Failed Implantation OR Repeat Failed Implantation OR RIF OR Failed Cycle OR Recurrent Reproductive Failure OR Repeat Reproductive Failure OR "Reproductive Techniques"[Mesh] OR Reproductive Technique OR Reproductive Techniques OR Reproduction Technique OR Reproduction Techniques OR Reproduction Technic OR Reproduction Technics OR Reproductive Technology OR Reproductive Technologies OR "Reproductive Techniques, Assisted"[Mesh] OR Assisted Reproductive Technique OR Assisted Reproductive Techniques OR Assisted Reproductive Technic OR Assisted Reproductive Technics OR Assisted Reproductive Technology OR Assisted Reproductive Technologies)

Results: 3,697

Search fields: All fields

**Cochrane :**

(sequential transfer OR sequential embryo transfer OR consecutive embryo transfer OR sequential embryo transplantation OR sequencing embryo transfer OR interval double transfer OR two-step transfer OR two-step embryo transfer OR Two-step consecutive transfer OR double embryo transfer OR sequential transplantation) AND (In Vitro Fertilization OR IVF OR In Vitro Fertilizations OR Test-Tube Fertilization OR Test-Tube Fertilizations OR Test Tube Fertilization OR Fertilizations in Vitro OR Test-Tube Babies OR Test Tube Babies OR Test-Tube Baby OR Recurrent in Vitro Fertilization Failure OR Recurrent in-vitro Fertilization Failure OR Recurrent IVF failure OR Recurrent Failure of in vitro Fertilization OR Intracytoplasmic Sperm Injection OR Intracytoplasmic Sperm Injections OR ICSI OR Embryo Implantation OR Poor Implantation OR Implantation Failure OR Repeated Implantation Failure OR Recurrent Implantation Failure OR Recurrent Failure of Implantation OR Recurrent Failure to Implant OR Repeat Failure To Implant OR Recurrent Failed Implantation OR Repeat Failed Implantation OR RIF OR Failed Cycle OR Recurrent Reproductive Failure OR Repeat Reproductive Failure OR Reproductive Technique OR Reproductive Techniques OR Reproduction Technique OR Reproduction Techniques OR Reproduction Technic OR Reproduction Technics OR Reproductive Technology OR Reproductive Technologies OR Assisted Reproductive Technique OR Assisted Reproductive Techniques OR Assisted Reproductive Technic OR Assisted Reproductive Technics OR Assisted Reproductive Technology OR Assisted Reproductive Technologies)

Results: 1,078

Search fields: Title Abstract Keywords

**WOS:**

# TS=((sequential transfer OR sequential embryo transfer OR consecutive embryo transfer OR sequential embryo transplantation OR sequencing embryo transfer OR interval double transfer OR two-step transfer OR two-step embryo transfer OR Two-step consecutive transfer OR double embryo transfer OR sequential transplantation) AND (In Vitro Fertilization OR IVF OR In Vitro Fertilizations OR Test-Tube Fertilization OR Test-Tube Fertilizations OR Test Tube Fertilization OR Fertilizations in Vitro OR Test-Tube Babies OR Test Tube Babies OR Test-Tube Baby OR Recurrent in Vitro Fertilization Failure OR Recurrent in-vitro Fertilization Failure OR Recurrent IVF failure OR Recurrent Failure of in vitro Fertilization OR Intracytoplasmic Sperm Injection OR Intracytoplasmic Sperm Injections OR ICSI OR Embryo Implantation OR Poor Implantation OR Implantation Failure OR Repeated Implantation Failure OR Recurrent Implantation Failure OR Recurrent Failure of Implantation OR Recurrent Failure to Implant OR Repeat Failure To Implant OR Recurrent Failed Implantation OR Repeat Failed Implantation OR RIF OR Failed Cycle OR Recurrent Reproductive Failure OR Repeat Reproductive Failure OR Reproductive Technique OR Reproductive Techniques OR Reproduction Technique OR Reproduction Techniques OR Reproduction Technic OR Reproduction Technics OR Reproductive Technology OR Reproductive Technologies OR Assisted Reproductive Technique OR Assisted Reproductive Techniques OR Assisted Reproductive Technic OR Assisted Reproductive Technics OR Assisted Reproductive Technology OR Assisted Reproductive Technologies))

# Results: 3,057

Search fields: Topic

**Scopus :**

(TITLE-ABS-KEY(("sequential transfer" OR "sequential embryo transfer" OR "consecutive embryo transfer" OR "sequential embryo transplantation" OR "sequencing embryo transfer" OR "interval double transfer" OR "two-step transfer" OR "two-step embryo transfer" OR "Two-step consecutive transfer" OR "double embryo transfer" OR "sequential transplantation")) AND TITLE-ABS-KEY(("In Vitro Fertilization" OR "IVF" OR "In Vitro Fertilizations" OR "Test-Tube Fertilization" OR "Test-Tube Fertilizations" OR "Test Tube Fertilization" OR "Fertilizations in Vitro" OR "Test-Tube Babies" OR "Test Tube Babies" OR "Test-Tube Baby" OR "Recurrent in Vitro Fertilization Failure" OR "Recurrent in-vitro Fertilization Failure" OR "Recurrent IVF failure" OR "Recurrent Failure of in vitro Fertilization" OR "Intracytoplasmic Sperm Injection" OR "Intracytoplasmic Sperm Injections" OR "ICSI" OR "Embryo Implantation" OR "Poor Implantation" OR "Implantation Failure" OR "Repeated Implantation Failure" OR "Recurrent Implantation Failure" OR "Recurrent Failure of Implantation" OR "Recurrent Failure to Implant" OR "Repeat Failure To Implant" OR "Recurrent Failed Implantation" OR "Repeat Failed Implantation" OR "RIF" OR "Failed Cycle" OR "Recurrent Reproductive Failure" OR "Repeat Reproductive Failure" OR "Reproductive Technique" OR "Reproductive Techniques" OR "Reproduction Technique" OR "Reproduction Techniques" OR "Reproduction Technic" OR "Reproduction Technics" OR "Reproductive Technology" OR "Reproductive Technologies" OR "Assisted Reproductive Technique" OR "Assisted Reproductive Techniques" OR "Assisted Reproductive Technic" OR "Assisted Reproductive Technics" OR "Assisted Reproductive Technology" OR "Assisted Reproductive Technologies")))

# Results: 4,65

Search fields: TITLE-ABS-KEY

--------------------------------------------------------------------------------------


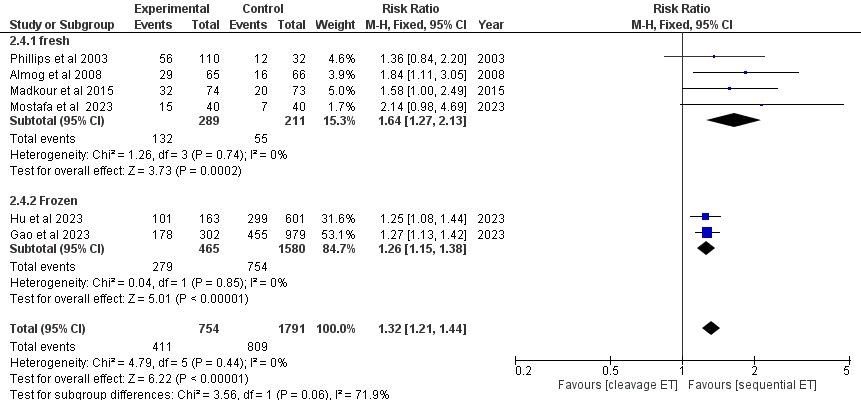


Supplementary Fig. 1. Forrest plot comparing chemical pregnancy rate between the sequential embryo transfer group and cleavage embryo transfer group.


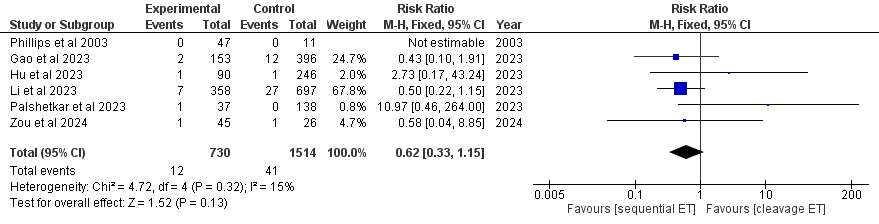


Supplementary Fig. 2. Forrest plot comparing ectopic pregnancy rate between the sequential embryo transfer group and cleavage embryo transfer group.


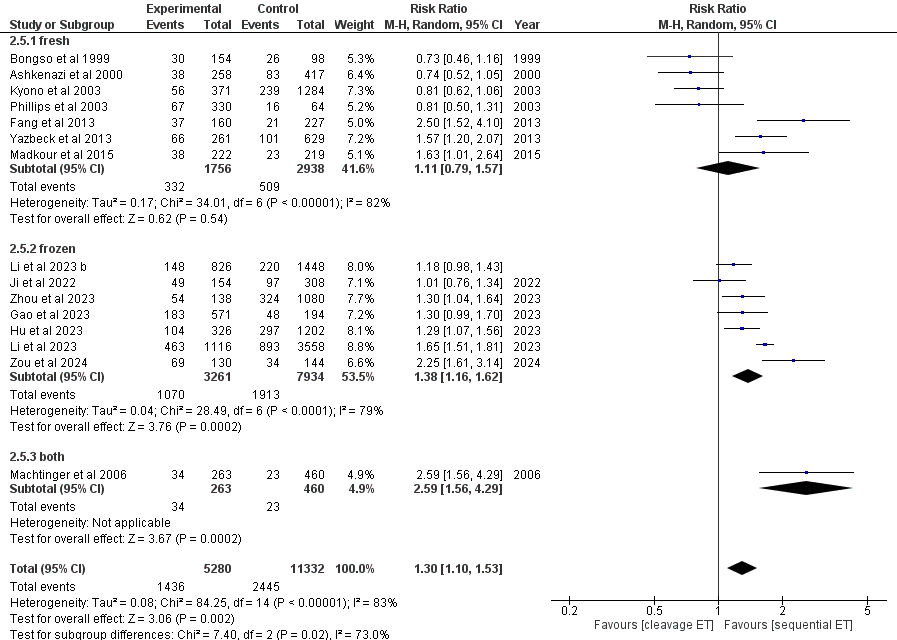


Supplementary Fig. 3A. Forrest plot comparing implantation rate between the sequential embryo transfer group and cleavage embryo transfer group.


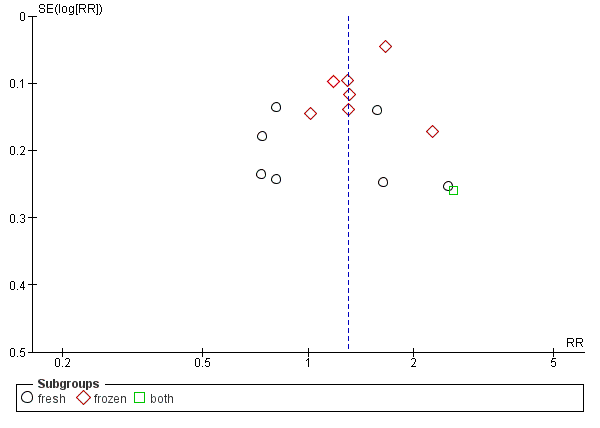


Supplementary Fig. 3B. Funnel plot of implantation rate.


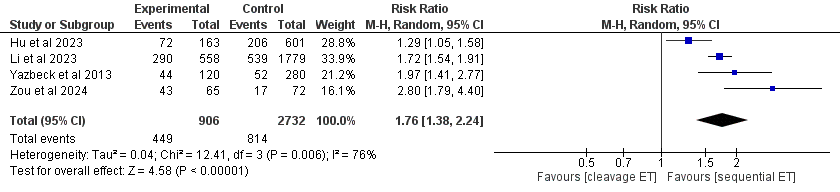


Supplementary Fig. 4. Forrest plot comparing live birth rate between the sequential embryo transfer group and cleavage embryo transfer group.


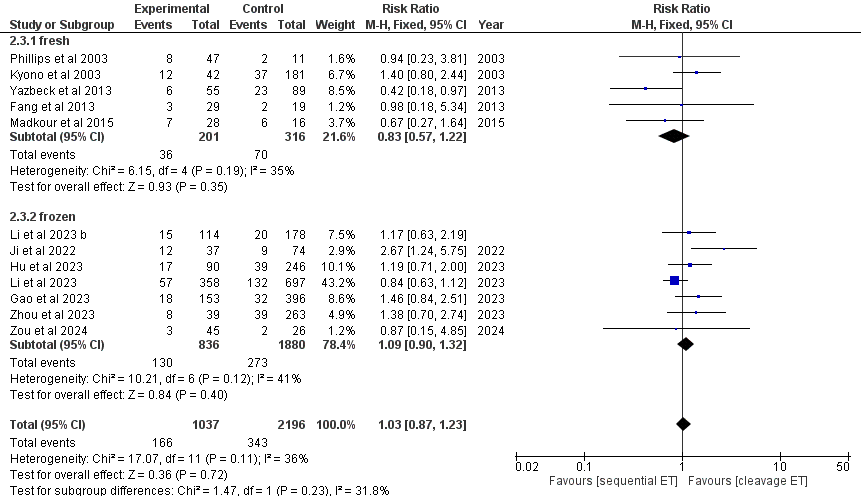


Supplementary Fig. 5 A. Forrest plot comparing miscarriage rate between the sequential embryo transfer group and cleavage embryo transfer group.


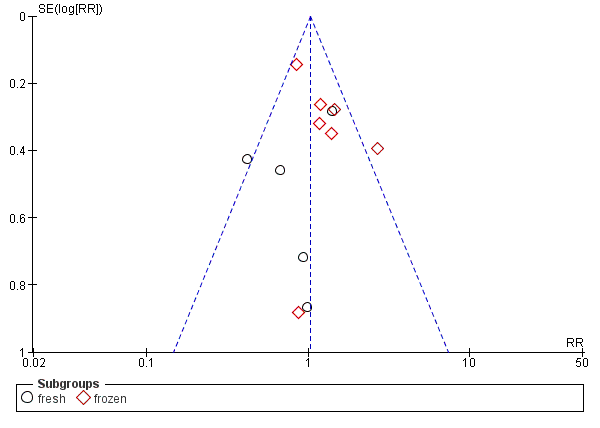


Supplementary Fig. 5B. Funnel plot of miscarriage rate.


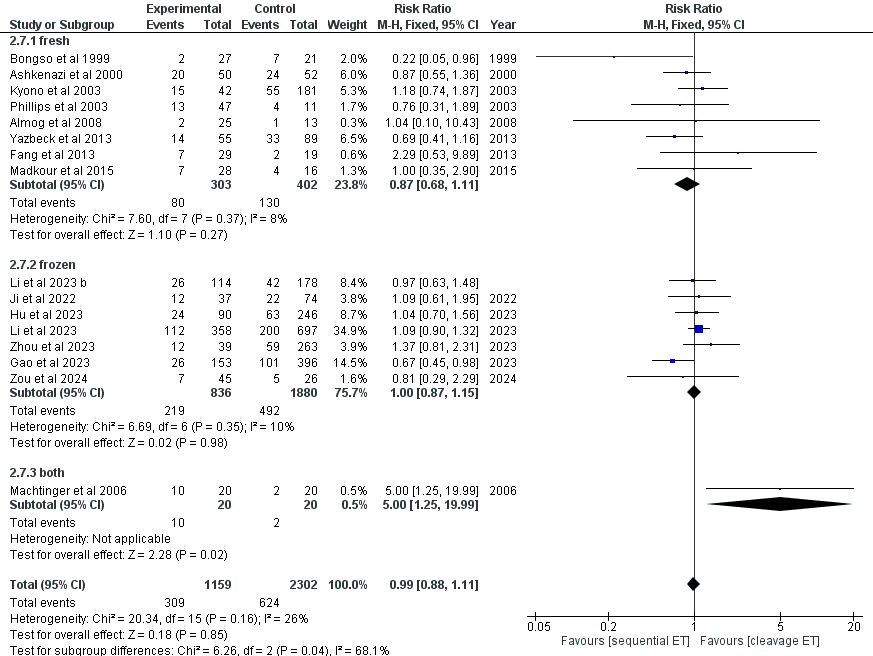


Supplementary Fig. 6a. Forrest plot comparing multiple pregnancy rate between the sequential embryo transfer group and cleavage embryo transfer group.


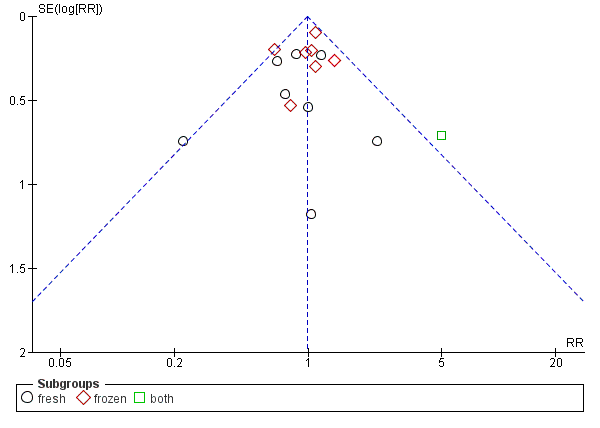


Supplementary Fig. 6b. Funnel plot of multiple pregnancy rate


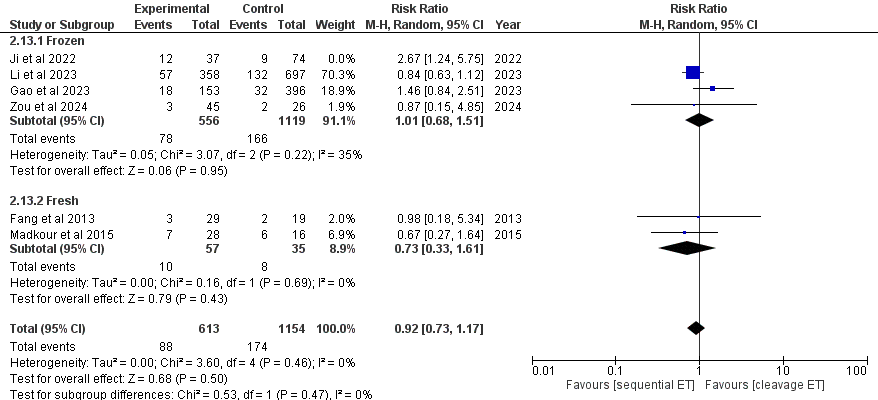


Supplementary Fig. 7. Forrest plot comparing miscarriage rate between the sequential embryo transfer group and cleavage embryo transfer group in patients with RIF.


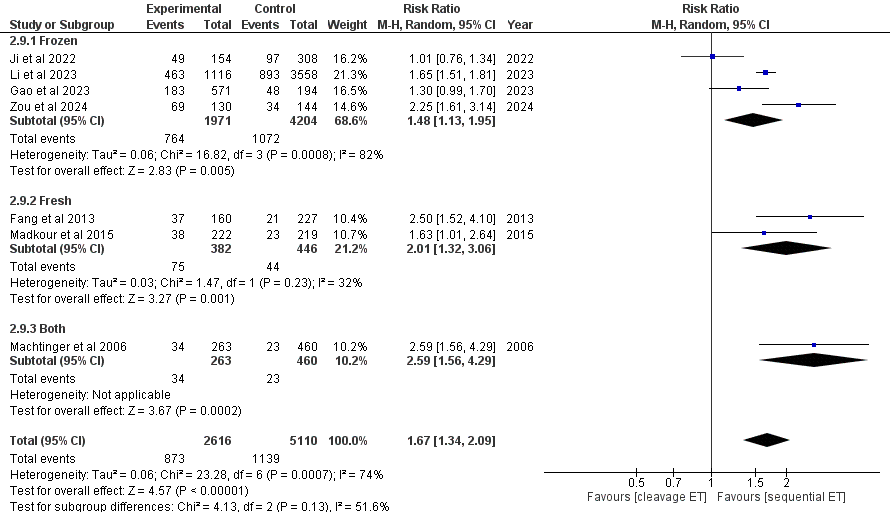


Supplementary Fig. 8. Forrest plot comparing implantation rate between the sequential embryo transfer group and cleavage embryo transfer group in patients with RIF.


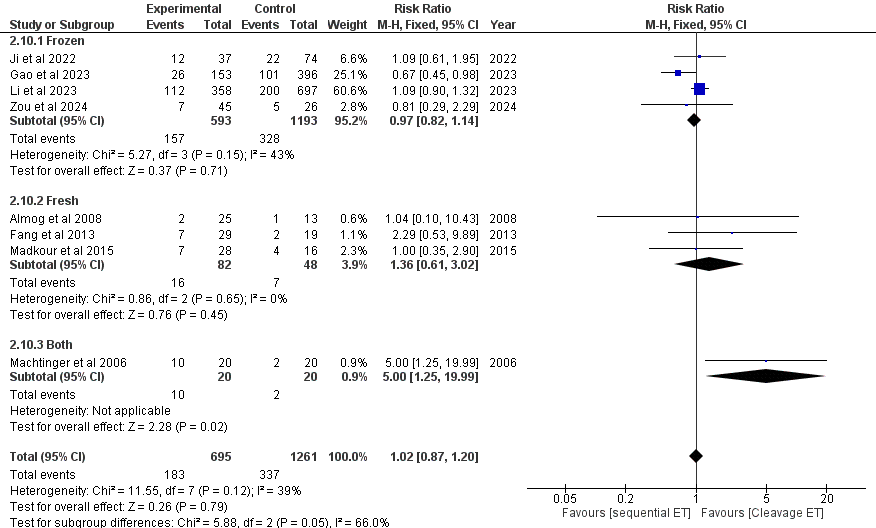


Supplementary Fig. 9. Forrest plot comparing multiple pregnancy rate between the sequential embryo transfer group and cleavage embryo transfer group in patients with RIF


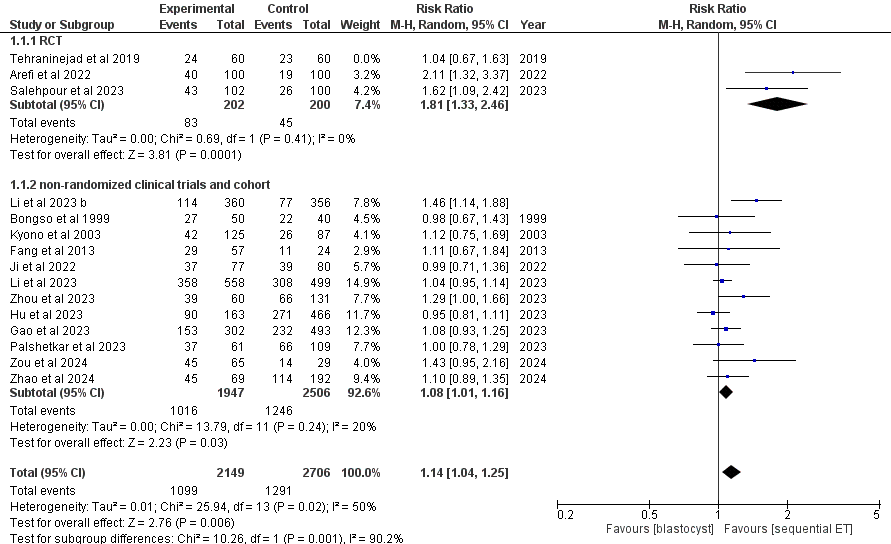


Supplementary Fig. 10. Forrest plot comparing clinical pregnancy rate according to the study design between the sequential embryo transfer group, and the blastocyst group.


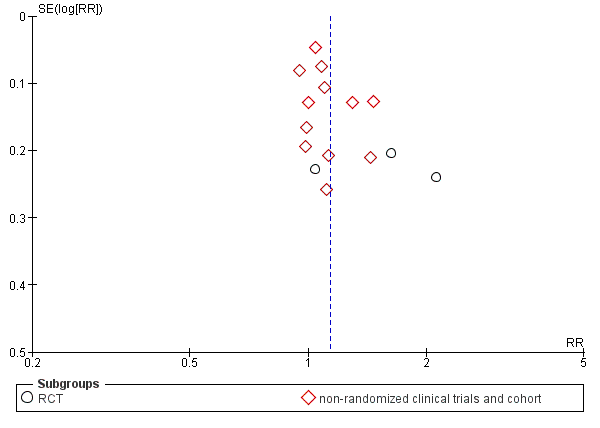


Supplementary Fig. 11. Funnel plot comparing clinical pregnancy.


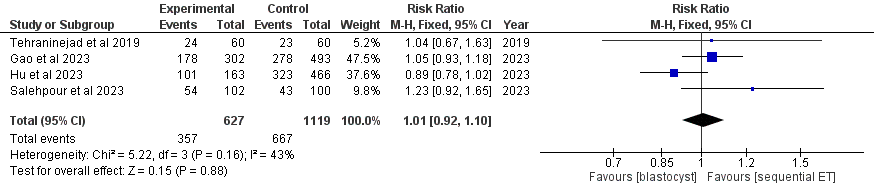


Supplementary Fig. 12. Forrest plot comparing chemical pregnancy rate between the sequential embryo transfer group, and the blastocyst group.


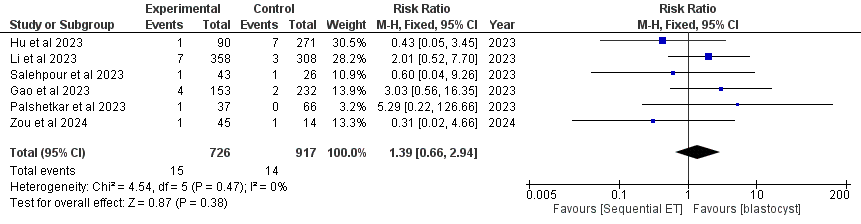


Supplementary Fig. 13. Forrest plot comparing ectopic pregnancy rate between the sequential embryo transfer group, and the blastocyst group.


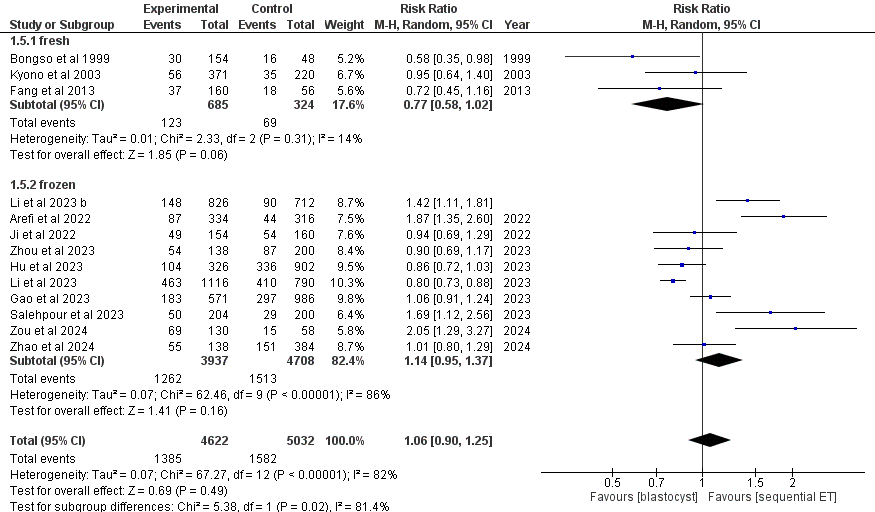


Supplementary Fig. 14A. Forrest plot comparing implantation rate between the sequential embryo transfer group, and the blastocyst group.


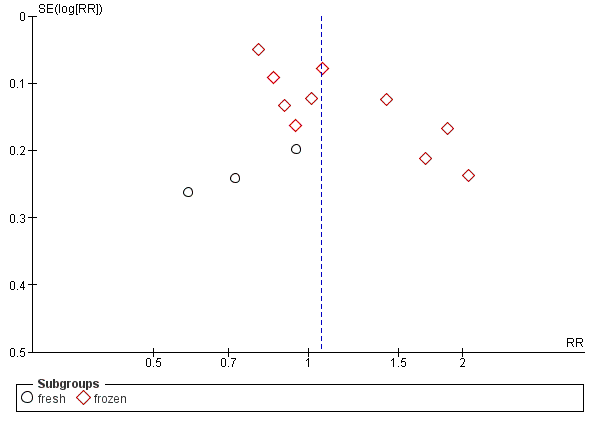


Supplementary Fig. 14B. Funnel plot of implantation rate.


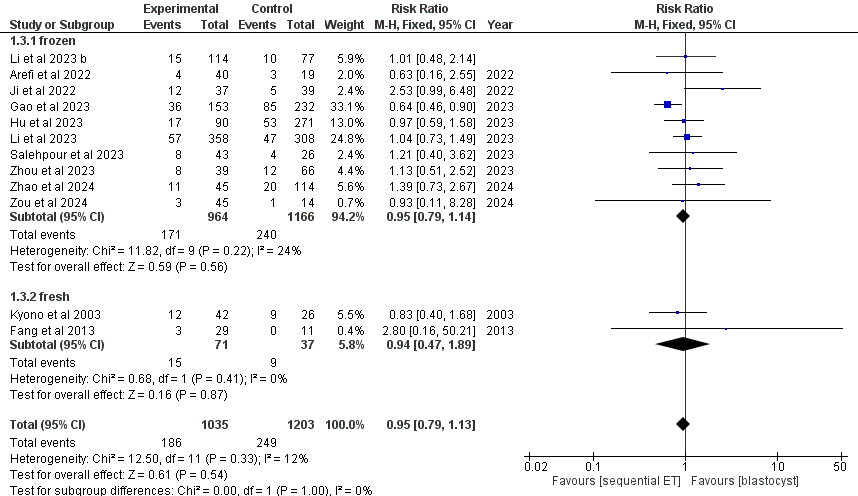


Supplementary Fig. 15. Forrest plot comparing miscarriage rate between the sequential embryo transfer group, and the blastocyst group.


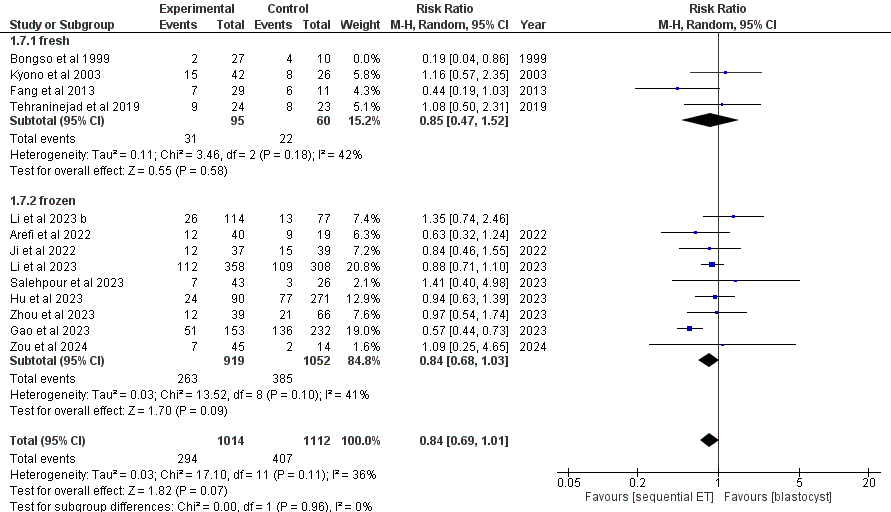


Supplementary Fig. 16 A. Forrest plot comparing multiple pregnancy rate between the sequential embryo transfer group, and the blastocyst group.


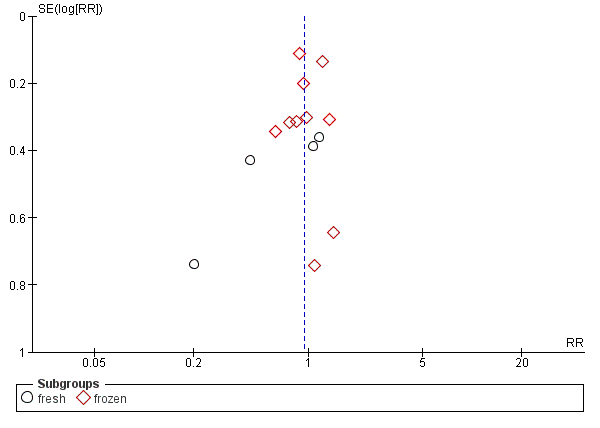


Supplementary Fig. 16B. Funnel plot of multiple pregnancy rate.


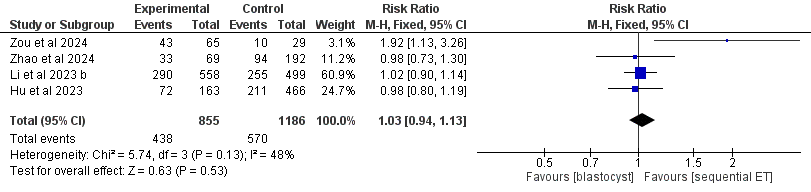


Supplementary Fig. 17. Forrest plot comparing live birth rate between the sequential embryo transfer group, and the blastocyst group.


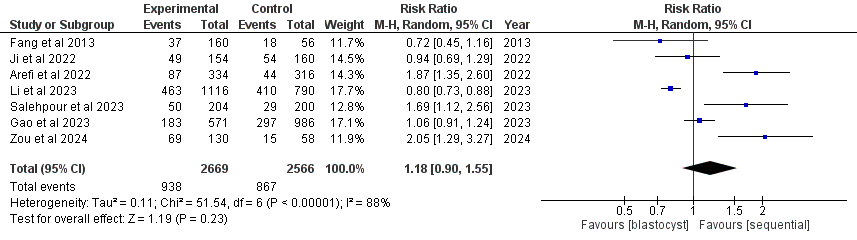


Supplementary Fig. 18. Forrest plot comparing implantation rate between the sequential embryo transfer group, and the blastocyst group in patients with RIF.


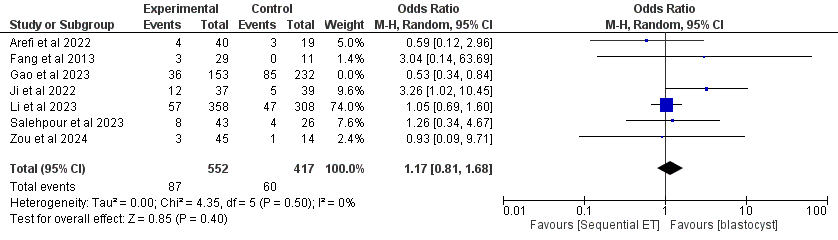


Supplementary Fig. 19. Forrest plot comparing miscarriage rate between the sequential embryo transfer group, and the blastocyst group in patients with RIF.


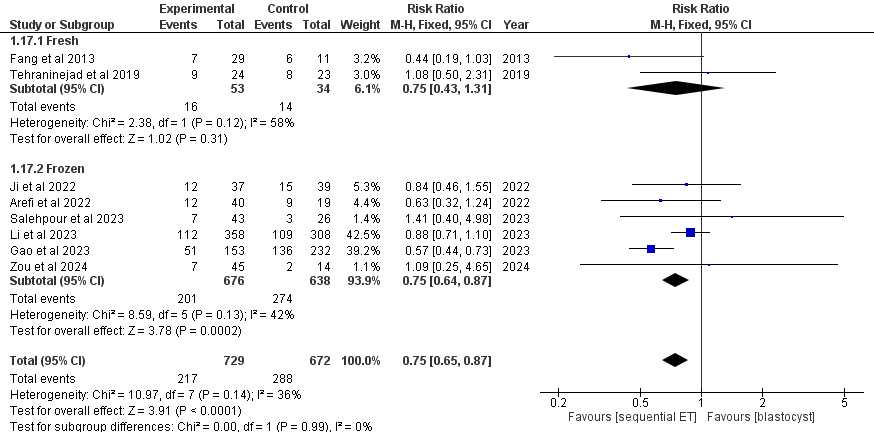


Supplementary Fig. 20. Forrest plot comparing multiple pregnancy rate between the sequential embryo transfer group, and the blastocyst group in patients with RIF.
